# The correlation between gut microbiome and atrial fibrillation: pathophysiology and therapeutic perspectives

**DOI:** 10.1186/s40779-023-00489-1

**Published:** 2023-11-07

**Authors:** Na Li, Ling Wang, Lei Li, Meng-Zhao Yang, Qing-Xiang Wang, Xi-Wen Bai, Feng Gao, Yi-Qiang Yuan, Zu-Jiang Yu, Zhi-Gang Ren

**Affiliations:** 1 Department of Infectious Diseases, the First Affiliated Hospital of Zhengzhou University, Zhengzhou, 450052 China; 2 Jinan Microecological Biomedicine Shandong Laboratory, Jinan, 250000 China; 3 Department of Cardiovascular Medicine, Henan Provincial Chest Hospital, Zhengzhou, 450008 China; 4 Department of Blood Collection, Xuchang Blood Center, Xuchang, 461000 Henan China; 5 Nanchang University Queen Marry School, Nanchang, 330036 China

**Keywords:** Atrial fibrillation (AF), Gut microbiome, Meta-omics, Metabolites, Immunity

## Abstract

Regulation of gut microbiota and its impact on human health is the theme of intensive research. The incidence and prevalence of atrial fibrillation (AF) are continuously escalating as the global population ages and chronic disease survival rates increase; however, the mechanisms are not entirely clarified. It is gaining awareness that alterations in the assembly, structure, and dynamics of gut microbiota are intimately engaged in the AF progression. Owing to advancements in next-generation sequencing technologies and computational strategies, researchers can explore novel linkages with the genomes, transcriptomes, proteomes, and metabolomes through parallel meta-omics approaches, rendering a panoramic view of the culture-independent microbial investigation. In this review, we summarized the evidence for a bidirectional correlation between AF and the gut microbiome. Furthermore, we proposed the concept of “gut-immune-heart” axis and addressed the direct and indirect causal roots between the gut microbiome and AF. The intricate relationship was unveiled to generate innovative microbiota-based preventive and therapeutic interventions, which shed light on a definite direction for future experiments.

## Background

From the results of physiological, epidemiological, and omics-based studies, complemented by cellular and animal experiments, microbial communities might coordinate or modify a significant portion of environmental impacts on human health [1–3]. These microorganisms are jointly classified as the microbiota and comprise a vast array of bacteria, archaea, phages, eukaryotic viruses, and fungi that coexist in all body cavities and on human surfaces. The gut microbiome serves an instrumental role in digesting food, fostering host immunity, registering intestinal endocrine function and neural signaling, eliminating toxins, modifying drug action and metabolism, as well as generating numerous compounds influencing the host [4–6].

Atrial fibrillation (AF) is the terminal ordinary endpoint of atrial remodeling attributable to multiple cardiac diseases and is a prominent contributor to cardiovascular morbidity and mortality [7]. Although the development of AF ablation techniques has created more alternatives for AF treatment, investigations have highlighted that nearly 50% of patients still demand repeat ablation [8]. Nowadays, the pathogenesis of AF has not been thoroughly elaborated, so it is pivotal to explore the underlying mechanism to prevent and manage it. Several recent researches have revealed that intestinal flora dysbiosis could induce intestinal barrier function impairment and systemic inflammatory response that contribute to atrial electrical and structural remodeling, ultimately contributing to the onset and development of AF [9–11]. The metabolites of intestinal flora, such as trimethylamine oxide (TMAO), short-chain fatty acids (SCFAs), and lipopolysaccharides (LPS), equally participate in AF occurrence.

To date, a search of PubMed (https://pubmed.ncbi.nlm.nih.gov/, accessed on August 2, 2023) using the search strategy (microbiome OR microbiota OR microbe) AND (atrial fibrillation) returned over 79 relevant published articles of which almost 28% were reviewed. Although there are some excellent recent reviews on this topic [12–14], we pioneered the concept of the gut-immune-heart axis and elaborated that intestinal microecological disorders could contribute to AF development through the axis. Modulating the composition and metabolism of the gut microbiome could serve as targets for therapeutic intervention in AF. We delivered an integrated overview of the literature on the correlation between gut microbiome’s clinical profile and AF, the experimental and clinical evidence specifying the basic mechanisms, as well as the status of various approaches for AF prevention and management. Furthermore, we addressed the present challenges to demonstrate causality, discussed the gaps in knowledge, and proposed a potential role for meta-omics technologies in uncovering these. In conclusion, this body of work reinforced the notion that gut microbiome might shed light on AF biomarkers and therapeutic interventions.

## Meta-omics for human microbiota characterization

**Fig. 1:**
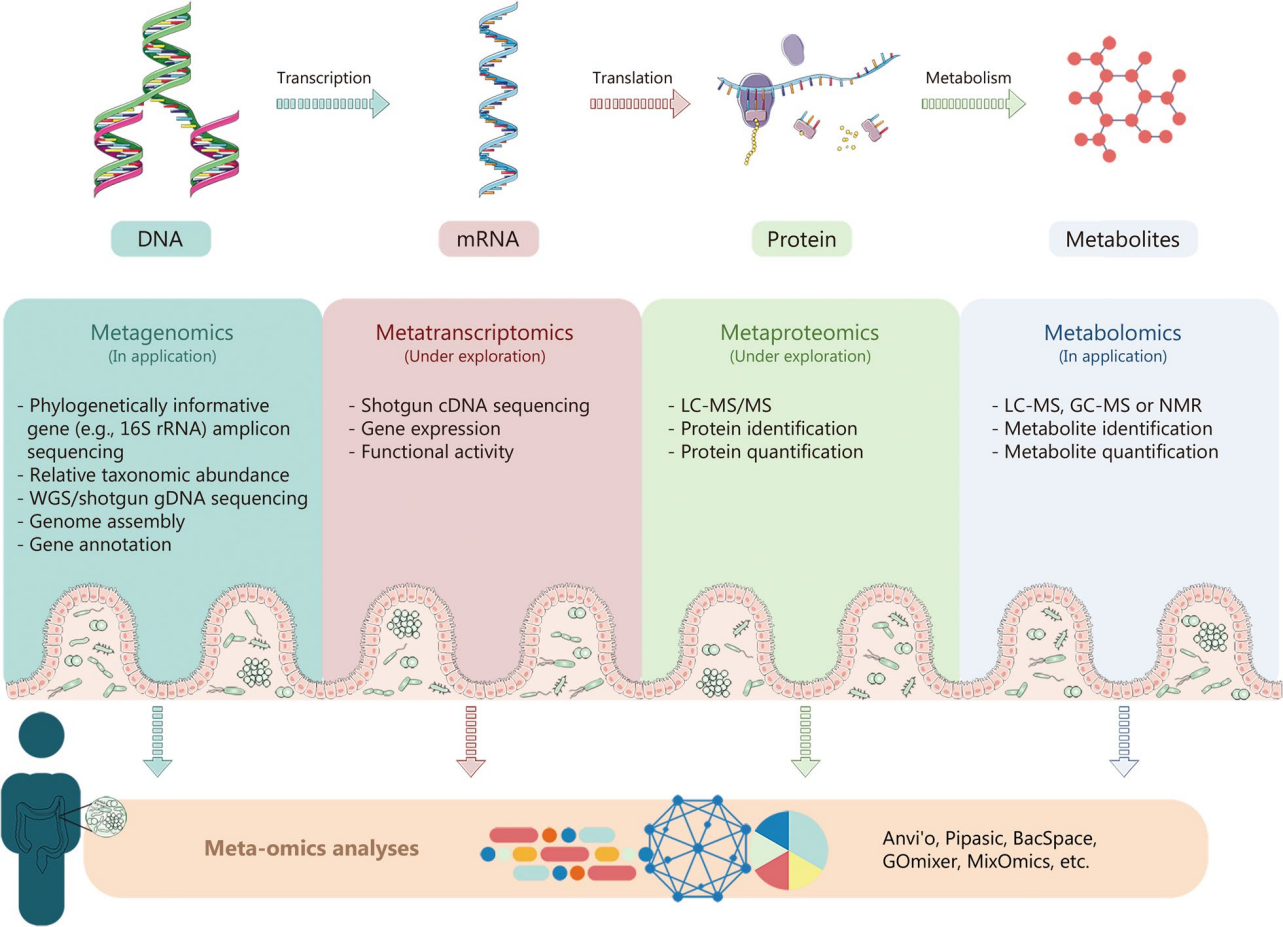
Meta-omics for host-microbiome interactions. Consolidation of data from meta-omics approaches provides expanded insights into microbiome capabilities, including metagenomics, metatranscriptomics, metaproteomics, and metabolomics. Currently, the most extensively practiced of the four omics are metagenomics and metabolomics. A cluster of pipelines for the proper execution of meta-omics research is presented, which inspects diverse aspects of the gut ecosystem at multiple levels with their advantages. GC gas chromatography, LC liquid chromatography, MS mass spectrometry, NMR nuclear magnetic resonance, WGS whole genome sequencing, gDNA genomic DNA, cDNA complementary DNA

Given the high complexity of the human microbiome and the difficulties in cultivating a high fraction of gut microbial species [15], the majority of microbiome investigations adopt “meta-omics” approaches, including metagenomics, metatranscriptomics, metaproteomics and metabolomics (Fig. 1). Currently, the most extensively practiced of the four omics are metagenomics and metabolomics. Hereby, we recapitulated a cluster of pipelines for the proper execution of meta-omics research, where inspected diverse aspects of the gut ecosystem at multiple levels with their advantages and disadvantages.

Metagenomic analyses deliver a precise and quantified portrayal of microbial composition. It is currently grounded in next-generation sequencing technology, which can be implemented for whole genome sequencing (shotgun metagenomics) or a single amplicon (16S rRNA gene sequencing). Targeted amplicon sequencing is a common method to detect sequence differences in bacterial 16S rRNA hypervariable regions for taxonomic identification of bacteria present. Regions of variability with 16S analyses, while facile and informative, are ordinarily insufficient to render species-level resolution. In addition, according to the different regions analyzed, varying outcomes can be yielded. Whole genomic shotgun sequencing employs high-throughput genomic sequencing in combination with sophisticated computational bioinformatics to capture profiles that identify microbial communities of both known or unknown taxonomy and underlying functions without the presence of laboratory cultures [16]. Although theoretically comprehensive and insightful, most surveys remain underpowered. The outcomes of such analyses are routinely stated as proportions rather than absolute levels, and the existence of specific microbes in a specimen may not equal pathogenicity. Notably, bioinformatics analysis of metagenomics sequences permits observers to characterize the key metabolic routes and functional components conducted by entire communities at the gene level [17]. It is imperative to reinforce that functional profiling at the genetic scale is still in its infancy, which furnishes licensed contacts between microbial organic composition and metabolic potential, rather than metabolic activity. This entails combining metagenomic data with other meta-omics analyses.

With nuclear magnetic resonance spectroscopy and mass spectrometry, metabolomics can be adapted for targeted analysis of a panel of identified compounds such as carbohydrates, lipids, and amino acids or for untargeted analysis (also referred to as “metabolic fingerprinting”) to enable robust metabolic comparison [18]. Identification of fecal metabolomes or targeted analysis of metabolites in AF and post-ablation AF can shed valuable light on bidirectional AF-microbiome interaction. Metabolomics has analogous weaknesses to incomplete databases and continues to be problematic in disentangling metabolites of host or microbiome origin and in linking metabolites to specific taxa [19]. Assays for metatranscriptomic studies typically involve reverse transcription and complementary DNA sequencing of RNA isolated from microbiomes of high quality and sufficient quantity. Community-level measurements of gene expression provide information about the patterns of gene expression that are induced or repressed under different circumstances [20]. Nevertheless, due to the pervasive prevalence of RNases in host-derived samples, it imposes challenges to dealing with laborious and complex procedures including sample collection, storage, and preparation when performing metatranscriptomic analyses [21]. Compared to metatranscriptomics, metaproteomics in principle affords deeper insight into gut microbial function, as not all transcripts are subliminally translated into proteins. However, metaproteomics is not quite a well-established field and suffers from various imperfections, including the absence of prevalent guidelines and protocols for performing metaproteomic tests and interpreting metaproteomic inclusions [22].

Consolidation of data from meta-omics approaches provides expanded insights into microbiome capabilities, elucidating gene regulation and coupling the appearance of microbes to metabolites. Metabolomics is incrementally merged with metagenomics to uncover patterns of covariation between microbiota composition/function and metabolites, which can also characterize the phylogenetic-specific contributions of metabolite generation. However, metatranscriptomics and metaproteomics have only been applied in a very small number of the latest investigations related to intestinal flora [23, 24]. Correlation analysis and multivariate statistical methods are the most direct and frequently utilized approaches to statistically investigate intercorrelations and identify salient features in multi-omics data integration [25, 26]. In addition, the availability of cutting-edge machine learning pathways may revolutionize our capability to interpret and integrate meta-omics data, facilitating the transformation of studies into mechanisms [27]. Overall, although meta-omics data integration remains provocative, the assemblage of multiple meta-omics datasets presents a prodigious alternative to thoroughly characterize the function, composition, and metabolic behavior of the microbiome.

## Profile of AF-related gut microbiota and the metabolites

Currently, only a few observational studies in small cohorts have reported variations of gut microorganisms in AF patients and patients with AF after catheter ablation [28–38] (Table 1). These transformations included a significant increase in species richness and diversity, a pronounced expansion of opportunistic pathogenic bacteria, and a distinct diminution of symbiotic bacteria. However, another Japanese publication on the gut microbial composition and dietary habits of AF ablation patients revealed contrasting results, with AF patients having lower gut microbial richness and no differences in diversity [36]. This could be attributed to the fact that most AF patients took proton-pump inhibitors to prevent esophageal ulcers before ablation, which would decrease the abundance and diversity of intestinal flora [39]. Moreover, the shift in gut microbiota and metabolic profiles was correlated with recurrent risk [31] and developed dynamically during AF progression [29, 30], which required validation in longitudinal cohorts. In addition, Fang et al. [37] recently identified that gut flora disequilibrium and microbial dysfunction were implicated in an exacerbated thromboembolic risk of AF. The above research was a predominantly cross-sectional survey from single regional population cohorts. The presence of diet, exercise, or medications [e.g., proton-pump inhibitor or anticoagulant therapy (dabigatran or rivaroxaban)] on the variability of gut microbiome was not collected and corrected, and no clear cause-effect relationship could be demonstrated. Well-designed multi-center, large-sample clinical studies are warranted to further verify these findings.

**Table 1:** Reported alterations in gut microbes and metabolites in various AF cohorts

Author	Subjects	Technique	Associated microbiota changes	Typical metabolites
Zuo et al. [28]	AF (*n* = 50); controls (*n* = 50)	Metagenomic and metabolomic analyses	AF compared with controls: Elevation in microbial diversity Increased: *Ruminococcus*, *Streptococcus*, and *Enterococcus* Decreased: *Faecalibacterium*, *Alistipes*, *Oscillibacter*, and *Bilophila*	AF compared with controls: Decreased: cholic acid, oleic acid, linoleic acid, and α-linolenic acid
Zuo et al. [29]	psAF: Persistence > 12 months (*n* = 8); Persistence < 12 months (*n* = 12); controls (*n* = 20)	Metagenomic and metabolomic analyses	psAF compared with controls: Elevation in microbial diversity Increased: *Blautia*, *Dorea*, and *Coprococcus* Decreased: *Butyricicoccus* and *Paraprevotella*	psAF compared with controls: Increased: stearamide, octadecanedioic acid, and lysophosphatidylcholine Decreased: oleic acid, choline, and some amino acids
Zuo et al. [30]	PAF (*n* = 30); psAF (*n* = 20); controls (*n* = 50)	Metagenomic and metabolomic analyses	AF (including PAF and psAF) compared with controls: Increased: *Ruminococcus* and *Streptococcus* Decreased: *Prevotella copri* and *Prevotella copri* CAG:164	AF (including PAF and psAF) compared with controls: Increased: chenodeoxycholic acid Decreased: α-linolenic acid
Li et al. [31]	RAF (*n* = 17); non-RAF (*n* = 23); controls (*n* = 50)	Metagenomic and metabolomic analyses	AF (including non-RAF and RAF) compared with controls: Elevated gut microbial diversity Increased: *Ruminococcus*, *Blautia*, *Dorea* and *Dialister* Decreased: *Prevotella*	AF (including non-RAF and RAF) compared with controls: Increased: lysophosphatidylethanolamine, chenodeoxycholic acid and sebacic acid Decreased: α-linolenic acid
Xu et al. [32]	A Chinese population with 1475 participants	16S rRNA	Increased: *Burkholderiales* and *Alcaligenaceae* Decreased: *Lachnobacterium*, *Bacteroides coprophilus*, *Barnesiellaceae*, an undefined genus in the family *Veillonellaceae* and *Mitsuokella*	–
Tabata et al. [36]	AF ablation patients (*n* = 34); controls (*n* = 66)	16S rRNA	AF compared with controls: Richness was lower; diversity did not differ Increased: *Parabacteroides*, *Lachnoclostridium*, *Streptococcus*, and *Alistipes* Decreased: *Enterobacter*	–
Huang et al. [33]	AF ablation patients (*n* = 36); controls (*n* = 30)	16S rRNA high-throughput sequencing and nontargeted metabolomic detection	AF compared with controls: Richness and diversity increased Increased: opportunistic pathogenic bacteria, such as *Klebsiella*, *Haemophilus*, *Streptococcus*, and *Enterococcus* Decreased: symbiotic bacteria, such as *Agathobacter* and *Butyrivibrio*; After catheter ablation: Species richness and diversity did not change significantly in the short-term Increased: symbiotic bacteria (*Lactobacillus*, *Agathobacter*, *Lachnospira*, etc.) Decreased: pathogenic bacteria (*Ruminococcus*, etc.)	AF compared with controls: Decreased: caffeine After catheter ablation: Increased: citrulline Decreased: oleanolic acid
Fang et al. [37]	High thromboembolic risk [CHA2DS2-VASc score ≥ 2 (males) or CHA2DS2-VASc score ≥ 3 (females)] (*n* = 32); low thromboembolic risk [CHA2DS2-VASc score < 2 (males) or CHA2DS2-VASc score < 3 (females)] (*n* = 18)	Metagenomic	High thromboembolic risk: Increased gut microbial diversity Increased: *Bacteroides* Decreased: *Prevotella*	–
Zuo et al. [38]	AF (*n* = 50); controls (*n* = 50)	Metagenomic	AF compared with controls: Elevated gut viral diversity Increased: *Secoviridae* and *Fimoviridae; Synechococcus phage S-SM1*, *Cronobacter phage CR5*, and *Staphylococcus phage SPbeta-like*	–
Palmu et al. [34]	Prevalent AF (*n* = 116); incident AF (*n* = 539); total individuals (*n* = 6763)	Metagenomic	No statistically significant differences in gut microbiome alpha or beta diversity Prevalent AF compared with total individuals: Increased: *Eisenbergiella*, *Enorma*, *Enterobacter*, and* Kluyvera* Decreased: *Bacteroides*, *Bifidobacterium, Holdemanella*, *Parabacteroides*, and *Turicibacter* Incident AF compared with individuals with prevalent AF excluded: Increased: *Bifidobacterium*, *Enorma*, *Lactococcus*, *Mitsuokella*, and* Sellimonas* Decreased: *Tyzzerella*, *Hungatella*, and *Sanguibacteroides*	–
Wang et al. [35]	POAF patients (*n* = 45); controls (*n* = 90)	16S rRNA	POAF compared with controls: The alpha diversity was higher in POAF patients Increased: *Lachnospira*, *Acinetobacter*, *Veillonella* and *Aeromonas* Decreased: *Escherichia-Shigella*, *Klebsiella*, *Streptococcus*, *Brevundimonas* and *Citrobacter*	POAF compared with controls: Decreased: plasma 25-hydroxy vitamin D

*AF* atrial fibrillation, *psAF* persistent atrial fibrillation, *PAF* paroxysmal atrial fibrillation, *POAF* post-operative atrial fibrillation, *RAF* recurrent atrial fibrillation

Modified gut microbial metabolites and associated metabolic patterns have also been visualized in AF samples. The potential bacteria and metabolic pathways that participated in trimethylamine (TMA) generation were significantly increased in the intestinal of AF patients [40]. Wang et al. [9] revealed that circulating LPS levels were identified as a predictor of AF recurrence. Moreover, it has been documented that enhanced serum TMAO or LPS levels foreshadowed adverse events in AF patients [41–43]. Nonetheless, in another study by Papandreou et al. [44], TMAO was unrelated to AF, contrary to its precursors (choline, betaine, and dimethylglycine). The outcomes of a Mendelian randomization study also demonstrated that high TMAO and carnitine were not linked to higher odds of AF after Bonferroni correction, possibly due to confounding or reverse causality [45]. Disruption of SCFAs synthesis-associated genes characterized by reduced harboring species and enzymatic genes as well as decreased levels of SCFAs in stool samples were identified in patients with AF [46, 47]. Increased levels of serum secondary bile acids (BAs), including glycocholate sulfate and glycocholate, were associated with the risk of AF [48, 49]. However, it was reported that AF patients exhibited dysregulated gut microbial biotransformation of primary to secondary BAs and a decreased proportion of secondary BAs in the stool [50]. These discoveries highlighted the critical role of intestinal bacteria in AF pathogenesis, thus demonstrating its therapeutic and diagnostic potential as an interventional strategy. Although these possibilities are compelling, they remain speculative pending direct evidence.

## Direct correlations between gut microbiome and AF

**Fig. 2:**
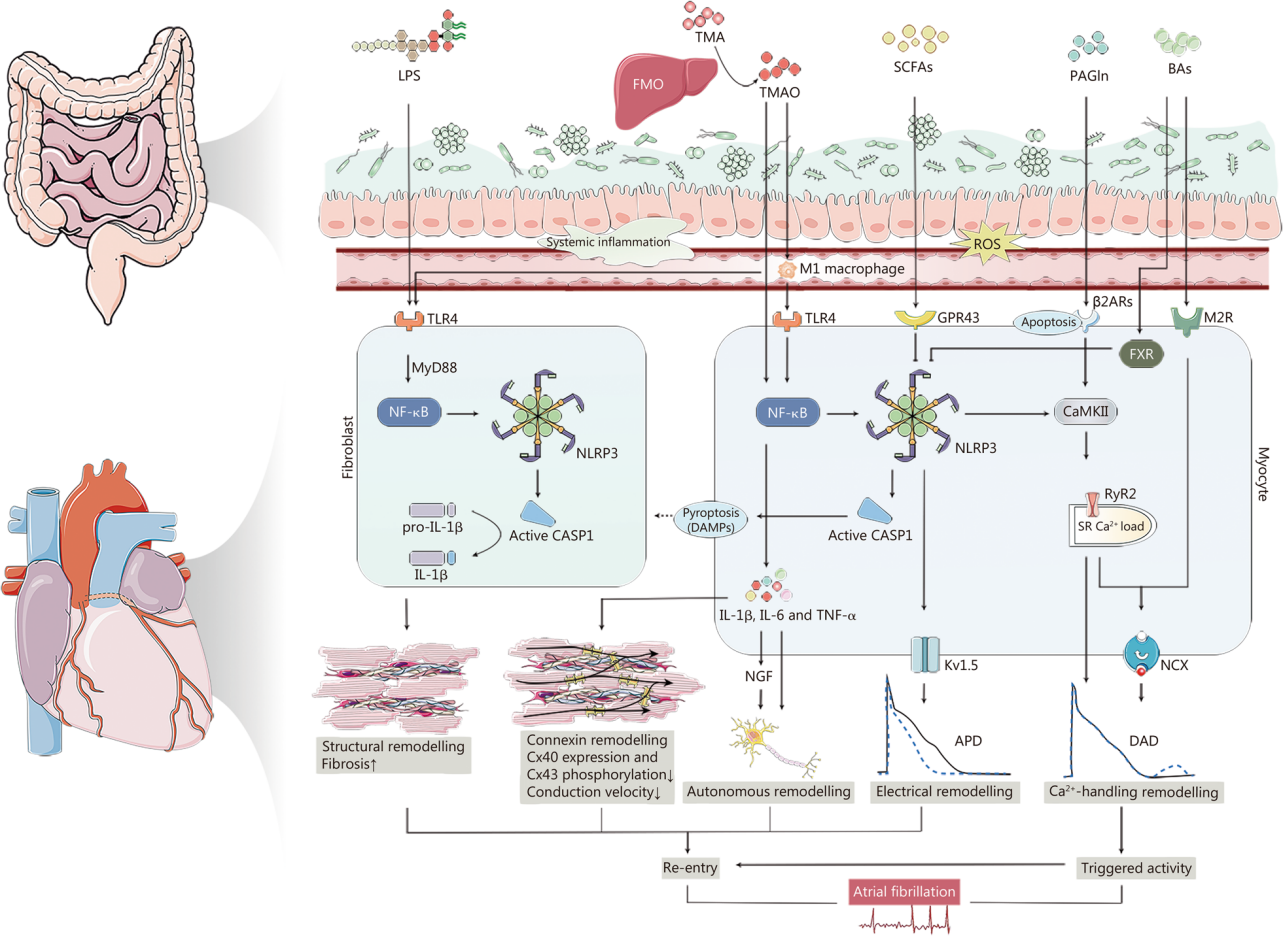
Molecular pathways of gut microbiome and the metabolites involved in atrial fibrillation (AF) progression. The pathogenesis of AF is ordinarily based on the substrate, including re-entry-promoting structures, connexin (Cx), and electrical remodeling, as well as Ca^2+^-handling remodeling facilitated by triggered activity. The TLR4/MyD88/NF-κB pathway primes and triggers the atrial NACHT, LRR, and PYD domains-containing protein-3 (NLRP3) inflammasome in response to lipopolysaccharides (LPS) stimulation, resulting in elevated secretion of downstream cytokines such as IL-1. Intestinal flora shape trimethylamine oxide (TMAO) synthesis, facilitate M1 macrophage polarization and pyroptosis and accentuate atrial structural remodeling. TMAO decreases Cx40 expression and Cx43 phosphorylation, possibly as a result of its contribution to increased infiltration of inflammatory cytokines such as IL-1β, IL-6, and tumor necrosis factor-α (TNF-α) in atrial tissue. Short-chain fatty acids (SCFAs) from intestinal symbionts attenuated NLRP3 signaling-mediated atrial fibrosis via GPR43 and downregulated the expression levels of phosphorylated calmodulin kinase II (CaMKII) and CaMKII-related ryanodine receptor 2 (RyR2) phosphorylation in the atria, thereby preventing Ca^2+^-handling disruption. Phenylacetylglutamine (PAGln) exacerbates oxidative stress and apoptosis and enhances activation of CaMKII and RyR2 in atrial myocytes by stimulating α2A, α2B and β2-adrenergic receptors (β2aRs). Bile acids (BAs) signaling via farnesoid X receptor (FXR) quench NLRP3 activation, whereas BA-induced Ca^2+^ influx can activate NLRP3 inflammasome. Moreover, discrepancies in levels of upstream factors, including the autonomic nervous system (ANS), systemic inflammation, and reactive oxygen species (ROS), can also interfere with both ectopic firing and re-entry-promoting substrate, thus directly contributing to the onset and evolution of AF. APD action potential duration, CASP1 caspase-1, DAD delayed afterdepolarizations, DAMPs damage-associated molecular patterns, IL interleukin, M2R M2 receptor, NCX Na^+^/Ca^2+^ exchanger, NF-κB nuclear factor-κB, NGF nerve growth factor, SR sarcoendoplasmic reticulum, TLR Toll-like receptor, TMA trimethylamine, FMO flavin-containing monooxygenase, MyD88 myeloid differentiation primary response protein 88, GPR43 G-protein-coupled receptor 43, Kv voltage-gated potassium

Maintenance of arrhythmias typically relies on a “substrate”, referring to the mechanical, electrophysiological, and anatomical features of atria that perpetuate AF. The elaboration of this substrate ordinarily incorporates both structural and electrical elements of atrial remodeling. Structural remodeling denotes organizational variations in the architecture, comprising macroscopic (atrial enlargement) and microscopic (fibrosis), whereas electrical remodeling entails alterations in the identity of ion channels that mediate atrial myocardial activation and transmission. Over the last few years, there has been a variety of experimental evidence elucidating, from both a cellular and molecular perspective, how multiple metabolites derived from the intestinal flora could influence the structural and electrical remodeling of AF (Fig. 2).

### Structural remodeling

Gut microbiota dysbiosis enhanced susceptibility to AF and atrial fibrosis as an upstream factor through LPS-induced NLRP3 (NACHT, LRR, and PYD domains-containing protein-3) inflammasome activation. A novel etiological role of anomalous gut microbiota in the pathogenesis of age-related AF was recently demonstrated by Zhang et al. [10] using fecal microbiota transplantation (FMT) rat models. Young rats that were subjected to 6 weeks of FMT utilizing fecal samples collected from the aged AF rats exhibited a significant elevation in the expression levels of transform growth factor-β1 (TGF-β1) and α-smooth muscle actin (two fibrosis-related marker proteins) in the atria. This effect was attributed to the “priming” and “triggering” of atrial NLRP3 inflammasome via the toll-like receptor (TLR) 4/MyD88/nuclear factor-κB (NF-κB) pathway and an enhanced nuclear translocation of NF-κB phosphorylation in response to LPS stimulation. But one technical restriction of this study was the absence of authentication that the recolonized microbiota was resembling the donor groups. Similarly, Kong et al. [11] discovered a significant increase in LPS-producing microbiota, such as *Desulfovibrionaceae*, and activation of the signaling pathway in obesity-related AF mice models. More importantly, the administration of NLRP3-targeted inhibitors could revert fibrosis [10, 11, 51–53], presumably due to a decrease in the secretion of interleukin (IL)-1 and IL-18 family cytokines downstream. Pathological shifts of microbiota metabolites and the atria in clinical patients further confirmed that NLRP3 inflammasome was associated with the pathogenesis of AF [10]. In addition, prior investigation has shown that NLRP3 could also be engaged in AF progression through Ca^2+^-handling dysregulation [54], and further comprehensive works are warranted to straightforwardly address the function of LPS.

Intestinal flora shaped TMA and TMAO synthesis, facilitated M1 macrophage polarization and pyroptosis, accentuated atrial structural remodeling, and culminated in AF. Luo et al. [55] revealed the pathogenic ramifications of gut microbiota in cold-related AF. Cold exposure contributed to diminished *Akkermansia muciniphila*, which was accompanied by an increment in the syncretization of TMA and TMAO, the latter fostering cardiac pyroptosis [upregulated expressions of caspase-1 (CASP1)-p20 and cleaved-gasdermin D] and ultimately atrial structural remodeling. Intriguingly, in contrast to previous studies, TMAO enhanced M1 macrophage infiltration at physiological concentrations to evoke apoptosis in cardiac myocytes (CMs) and fibrosis in cardiac fibroblasts (CFs) rather than directly invigorating CFs [56]. Furthermore, emerging evidence underpinned the synergistic relationship between CMs and CFs in prompting a substrate for AF development [57]. CMs underwent pyroptosis in response to mature CASP1, resulting in the liberalization of danger-associated molecular factors (e.g., ATP, DNA) to initiate CFs, which also further stimulated cytokine release and collagen manufacture [54]. The sophisticated molecular mechanisms involved deserve deeper investigation. Preceding examinations have established that potentiated activation of NLRP3 inflammasome in CMs incremented CASP1 cleavage and CASP1-mediated pyroptosis, promoting the formation of an AF maintenance substrate [58]. Moreover, inhibition of gasdermin D with necrosulfonamide and conditional CASP1 knockout could forestall cold-related AF and reverse atrial fibrosis [55]. Therefore, whether NLRP3 inflammasome intermediates the pyroptosis mechanism in the TMAO pro-AF effect is a promising line of research.

Dietary fiber-fermented SCFAs from intestinal symbionts attenuated NLRP3 signaling-mediated atrial fibrosis and thus conserved against the prodrome of AF. It has been demonstrated that the administration of propionate depleted cardiac hypertrophy, fibrosis, arrhythmia susceptibility, and atherosclerotic lesion burden by altering T helper cell homeostasis [59]. Increased left atrial diameter and impaired ejection fraction were detected in mice fed a low-fiber diet, but not in mice fed a high-fiber diet or supplemented with SCFAs [47]. This was consistent with disorganized fibrosis and collagen expenditure displaying a striking progression in the atrial tissue of mice on a low-fiber diet. Thereafter, Zuo et al. [47] demonstrated that by autophagic degradation through K48- and K63-linked ubiquitylation, SCFAs could halt NLRP3 inflammasome activation via G protein-coupled receptor 43 (GPR43) in both mice and HL-1 cells, and thus exert a protective role on AF. However, the particular role of SCFAs in GPR43 expression and the bridge between SCFAs and NLRP3 mobilization still demand specific investigations.

Another gut microbiota-dependent metabolite, phenylacetylglutamine (PAGln), as an independent risk factor for AF, was positively correlated with left atrial enlargement [60]. PAGln stimulated α2A, α2B, and β2 adrenergic receptors [61], which could lead to the initiation and perpetuation of AF [62]. Fang et al. [60] have evaluated that PAGln aggravated oxidative stress and apoptosis in atrial myocytes and directly damaged them. Cardiomyocyte apoptosis inspired by CASP3 was correlated with the progression of AF by inquiring about atrial remodeling and declining conduction velocity [63]. Nevertheless, probably due to the small sample size, several results in this cross-sectional study were not statistically significant. Thus, additional large prospective cohort surveys and mechanistic studies are mandatory.

### Electrical/Ca^2+^-handling remodeling

Electrical remodeling yielded a substrate for facile reentry. The main dimensions of electrical remodeling in the atria encompassed shortening of the refractory period due to the downregulation of Ca^2+^ currents [64], accelerated repolarization and hyperpolarization of atrial cells owing to increased inward K^+^ currents [65], and conduction abnormalities because of altered expression and localization of connexins connecting atrial myocytes [66]. By definition, AF is a highly irregular atrial rhythm, and therefore electrical remodeling is paramount to the occurrence and sustenance of AF. Diverse metabolites of the intestinal flora could simultaneously exert an electrical remodeling that collectively augments AF susceptibility while imposing structural damage [67].

NLRP3 inflammasome signaling, a central proarrhythmic mediator of various pathophysiological signals in AF, probably contributed to Ca^2+^-handling abnormalities elicited by intestinal flora. Mice with constrained constitutive activation of cardiomyocytes with NLRP3 inflammasome exhibited elevated atrial ectopy due to upregulated ryanodine receptor 2 (RyR2) expression and abbreviated action potential duration that facilitated reentry (as atrial selective ultra-rapid delayed rectifier K^+^ currents and acetylcholine-activated inward rectifier K^+^ currents) [54]. It was demonstrated that replenishment of SCFAs downregulated the expression level of phosphorylated calmodulin kinase II (CaMKII) and CaMKII-related RyR2 phosphorylation in the atria, thereby preventing Ca^2+^-handling disruption coordinated by dietary fiber deprivation [47]. This interaction was mitigated by the alleviation of CaMKII phosphorylation through GPR43-mediated NLRP3 deactivation. Notably, no specific subunits or related molecules downstream of GPR43 and CaMKII were ascertained, necessitating further studies to comprehend the ramifications of pivotal agents in NLRP3 signaling. Zuo et al. [50] identified decreased secondary BAs and circulating fibroblast growth factor 19 levels in AF patients, leading to impaired protective function of lipid accumulation as well as signaling dysregulation in atrial cardiomyocytes. Taurine-bound primary BAs might precipitate alternations in membrane potential by activating Na^+^/Ca^2+^-exchanger and muscarinic M2-receptors in cardiomyocytes [68, 69]. BAs signaling via farnesoid X receptor has been demonstrated to quench NLRP3 activation, whereas BA-induced Ca^2+^ influx could activate NLRP3 inflammasome [70]. Alternatively, PAGln could potentiate the activation of CaMKII and RyR2 in atrial myocytes [60], facilitating cell membrane hyperexcitability and afterdepolarizations to instigate a proarrhythmic circumstance; however, the exact mechanism remains elusive.

Different metabolites of intestinal flora could alter the interpretation and dissemination of gap junctional proteins that compromise atrial electrical conduction. Gap junction ion channels like Cx40 and Cx43 mediated the electrical and metabolic coupling of cardiomyocyte-to-cardiomyocyte. Any modifications in connexin expression, phosphorylation, and distribution might result in enhanced refractoriness dispersion, heterogeneous repolarization, and reduced conduction velocity, culminating in the initiation and maintenance of AF [66, 71]. Jiang et al. [72] recently discovered that TMAO facilitated connexin remodeling, hallmarked by decreased Cx40 expression, reduced Cx43 phosphorylation, and lateral localization of both connexins. This might be attributed to enhanced infiltration of inflammatory cytokines such as IL-6, IL-1β, and tumor necrosis factor (TNF)-α in the atrial tissue by aberrant TMAO [73–76]. Abnormalities in connexins incremented the dispersion of the atrial effective refractory period and prompted AF sensitivity. Treatment with 3,3-dimethyl-1-butanol (DMB), a specific TMAO inhibitor, ameliorated atrial inflammation and further attenuated gap junction dysfunction. Analogously, the districting patterns of Cx43 and N-cadherin were pronouncedly revised and strongly lateralized by non-localized deposition in mice with low-fiber dietary, which could be alleviated by SCFAs supplementation [47]. Chen et al. [77] established that in human umbilical vein endothelial cells and aortas from ApoE^−/−^ mice, TMAO-triggered vascular inflammation was connected with the NLRP3 inflammasome activation. Nonetheless, no existing reports have addressed the potential role of NLRP3 inflammasome in the atrial inflammation invoked by TMAO.

### Autonomic nervous system (ANS), inflammation, reactive oxygen species (ROS) and microRNAs as direct mediators

Discrepancies in levels of upstream factors, including the ANS, inflammation, and ROS, could interfere with both ectopic firing and re-entry-promoting substrate. Intestinal flora and the metabolites could leverage these upstream factors and thus directly contribute to the onset and evolution of AF.

Gut microbes and their metabolites might influence the cardiac ANS through direct pathways (gut microbe-derived metabolites) and indirect pathways (gut-brain-heart axis). ANS was well recognized to play an overwhelming role in the pathogenesis and perpetuation of AF [78, 79]. Yet, the regulation of ANS-induced AF by intestinal flora is still in the preliminary stage of exploration. Yu et al. [80] revealed that after local TMAO injection to 4 major atrial ganglionated plexi (the anterior right, the inferior right, the superior left, and the inferior left ganglionated plexi), electrical and autonomous remodeling were noted in both normal canines and rapid atrial pacing-induced AF models. Moreover, TMAO acted through the cardiac ANS, exacerbating the malignant cycle of “AF begets AF”. A potential underlying mechanism for these effects might be the activation of the p65 NF-κB pathway, resulting in the uplift of pro-inflammatory cytokines such as TNF-α, IL-1β, and IL-6 [81], which probably meshed the neural activating properties of TMAO [82]. However, one limitation of this study was the lack of interventions to suppress the observed effects. Considering the intense relationship between heart and gut microbes, as well as the proximate attribution of microbiota to ANS [83–85], the gut-brain-heart axis might also have a regulatory role in AF progression. Still, this concept demands additional clinical and preclinical trials to be substantiated.

Dysbiosis of the intestinal flora could induce intestinal barrier function impairment and systemic inflammatory response, promoting atrial structural remodeling and electrical remodeling. Various metabolites of the intestinal flora have been described in detail to enhance AF susceptibility by generating manifold pro-inflammatory cytokines in atrial through signaling pathways such as NF-κB, NLRP3, TLR4, and so on. It was recently demonstrated that gut microbiota disorders could promote AF by exacerbating conduction disturbances and linoleic acid/sirtuin 1 signaling imbalances [67]. Furthermore, deterioration of the intestinal barrier function could trigger the entry of toxic bacteria metabolites into circulation and escalate the low-level inflammatory response of the circulatory system [86, 87]. Elderly and obese microbiota could result in loss of enterocyte microvilli and a considerable decline in the levels of intestinal mucosal barrier proteins (Claudin-1 and mucin 2) [10, 11]. It could be hypothesized that AF induced by aged and obese microbiota dysbiosis was affiliated with systemic inflammation. In response to LPS stimulation, paracrine action of inflammatory cytokines or delivery to distant organs might entail endotoxemia, which would precipitate atrial remodeling and consequently the occurrence of AF [9, 41, 88].

ROS was another conceivable bridge between turbulent intestinal flora and the pathogenesis of AF. Investigations have demonstrated that metabolites of gut microbiota, including LPS, SCFAs, indoxyl sulfate, and PAGln, might be intertwined with ROS synthesis [47, 60, 89]. ROS was an important stimulant to structural remodeling and electrical remodeling, acting through downstream systems like NLRP3-inflammasome formation, mitogen-activated protein kinases, and profibrotic inflammatory signaling [54, 90–92]. Besides, it has recently been recognized that ferroprotein-mediated ferroptosis was involved in the new onset of AF due to LPS-induced endotoxemia [11, 93]. As a type of oxidative death, ferroptosis was characterized by a high level of iron accumulation and lipid peroxidation. Suppression of CF-derived exosome miR-23a-3p to mitigate oxidative stress damage and ferroptosis could forestall AF progression toward persistence [94]. Indeed, only a few works in the literature have investigated the mechanisms by which ferroptosis exerts its role in the intestinal flora regulation of AF.

A burgeoning area in the AF pathophysiology was the contribution of microRNAs (miRNAs or miRs), tiny non-coding RNAs (approximately 22 nucleotides) that adversely regulated target genes. In tissue samples from AF patients, abnormal atrial autoregulation has been inferentially implicated in AF-related ectopy by altering hyperpolarization-activated cyclic nucleotide-gated channels expression and automaticity-modulating miRNAs [95], but direct evidence was scarce. Recent evidence has demonstrated that the microbiome could modulate colonic miR-155 or vascular miR-204 to influence cardiovascular motility [96, 97]. Nonetheless, whether miRNAs are involved in the mechanism of gut flora regulation of AF remains an open question.

## Indirect impact of the gut-immune-heart axis on AF

**Fig. 3:**
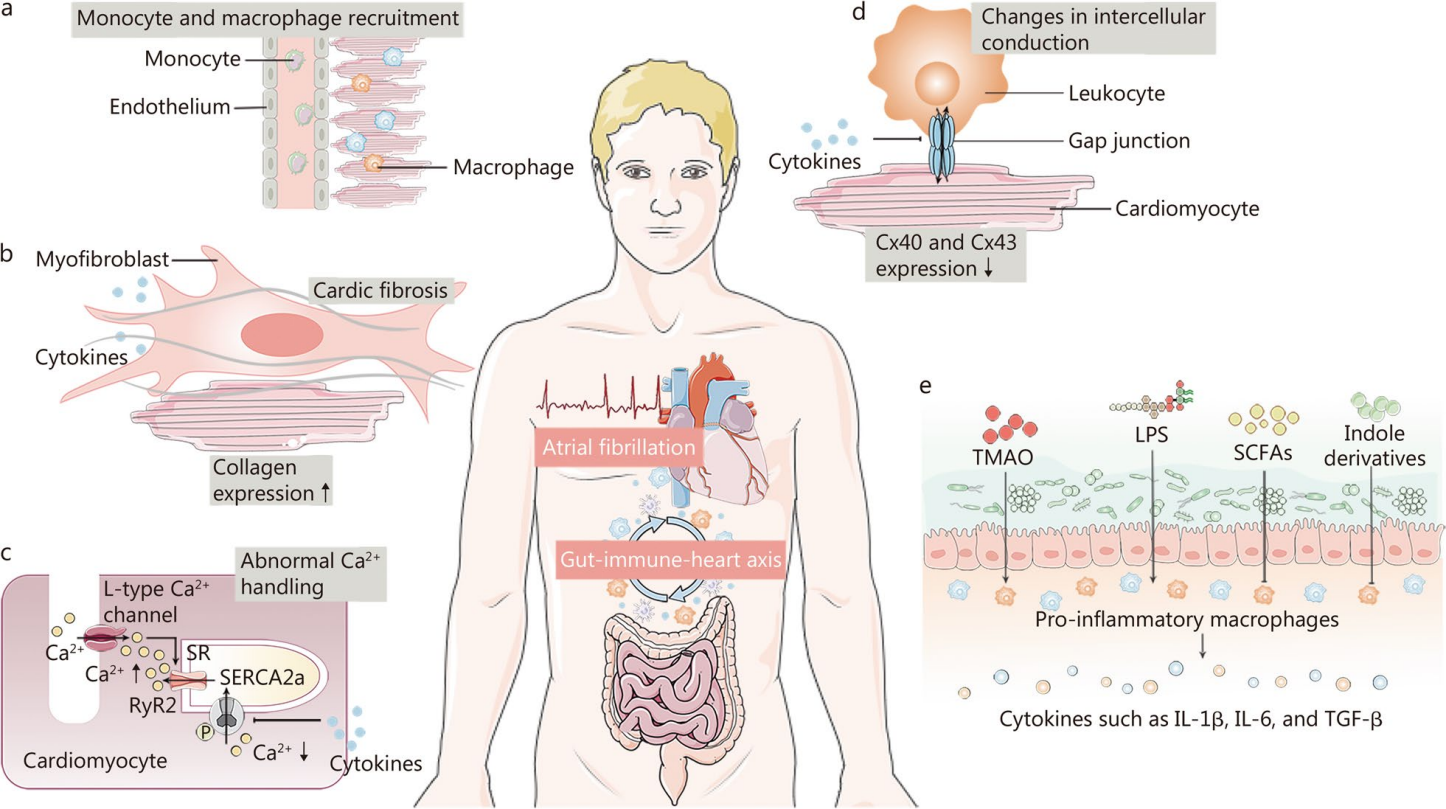
Gut-immune-heart axis. The gut microbiome can manipulate the immune system to control risk factors and indirectly induce the pathology of atrial fibrillation (AF) through the gut-immune-heart axis. **a** In the lesioned heart, monocytes and macrophages undergo phenotypic alterations and are massively recruited to the injury site, impacting the cardiomyocyte action potential by regulating repolarization, conduction velocity, and heterogeneity. **b** Macrophages can trigger the transformation of cardiac fibroblasts to myofibroblasts by releasing cytokines, leading to collagen deposition and facilitating structural remodeling. **c** Cytokines emitted by leukocytes can also influence ion channel expression (e.g., sarcoplasmic/endoplasmic reticulum Ca^2+^ ATPase 2a), which leads to abnormal Ca^2+^-handling in cardiomyocytes. **d** Cytokines released from leukocytes can impede intercellular conduction between cardiomyocytes and non-cardiomyocytes (e.g., leukocytes) by decreasing the expression of connexin (Cx) protein. **e** Microbiome is an emergent mediator of macrophage function and can influence the generation of pro-inflammatory cytokines by macrophages through metabolites (of which TMAO and LPS are promoters, while SCFAs and indole derivatives are inhibitors). LPS lipopolysaccharides, RyR2 ryanodine receptor 2, SCFAs short-chain fatty acids, SR sarcoplasmic reticulum, TMAO trimethylamine oxide, SERCA2a sarcoplasmic/endoplasmic reticulum Ca^2+^-ATPase 2a, Cx40 connexin 40, Cx43 connexin 43, IL interleukin, TGF-β transforming growth factor-β

The gut microbiota could indirectly be accountable for AF pathogenesis by manipulating the immune system to control AF risk factors. Efficacious measurement of AF risk factors was an imperative component of the comprehensive AF administration. Cardiovascular diseases such as hypertension [98], coronary artery disease [99], and heart failure [100] were considered the major risk factors for AF development. Intestinal flora disequilibrium could compromise the dysfunction and maturation of the immune system, thereby interfering with the performance of the cardiovascular system, which is the concept of the gut-immune-heart axis (Fig. 3). Potential indirect causal roots exist between gut microbiota and AF due to the interaction of microbial metabolites with the immune system.

### The role of immunity in cardiac arrhythmias

The various pathological mechanisms of multiple major cardiovascular risk factors might conflate in an ultimately shared avenue: expansion of leukocytes in the atrial myocardium, and contributing to the release of pro-inflammatory cytokines. The innate immune response is always launched by the discrimination of pathogen-associated molecular patterns (PAMPs) and/or damage-associated molecular patterns (DAMPs) by pattern recognition receptors (PRRs) and further induces an adaptive immune response. The main response triggered by PRRs binding of PAMPs or DAMPs is inflammation. According to the inflammatory and atypical function of cardiac immune cells, leukocytes could induce arrhythmias by interacting with cardiomyocytes or altering the composition of tissue. Monocytes and macrophages were the most plentiful leukocytes in the heart, which were recruited in enormous numbers to the site of injury in the atria of AF patients [101, 102] (Fig. 3a). Similar induction of AF was observed for macrophage-derived IL-1β and its downstream IL-6 [74, 103]. Moreover, macrophages could liberate cytokines to regulate the extracellular matrix and thus activate CFs to myofibroblasts, leading to collagen deposition and structural remodeling [104] (Fig. 3b). Upstream of cytokine release, NF-κB signaling and NLRP3 inflammasome resulted in arrhythmias. Additional studies have revealed that macrophages could generate matrix metalloproteinase 7 in the acute phase after myocardial infarction, a protease that could cleave extracellular matrix substrates [105, 106].

Immune cells might mediate conduction by modifying the maturation of the conduction system, adjusting the expression and function of ion channels in cardiomyocytes, interfering with gap junction communication between cardiomyocytes, as well as regulating turnover to quarantine the extracellular matrix. TNF released by leukocytes might affect Ca^2+^-handling in cardiomyocytes by electrical remodeling, such as by decreasing sarcoplasmic/endoplasmic reticulum Ca^2+^ ATPase 2a expression [107] (Fig. 3c). Notably, cardiomyocytes and leukocytes shared several ion channels, such as ORAI1 and its activator stromal interaction molecule 1. Cardiomyocyte-restricted stromal interaction molecule-knockdown mice displayed enhanced arrhythmia susceptibility triggered by discordant action potential duration alternans [108]. Considering the massive number of resident immune cells in the heart [109], how they may affect the depolarization or repolarization of cardiomyocytes and arrhythmias merits further study. In addition, direct cardiomyocyte-macrophage interaction via connexin (Cx) 40 and Cx43 containing gap junction was discerned in both mouse and human hearts [76, 110], which permitted electrical coupling between these two cell types (Fig. 3d). It has been suggested that macrophages might impact cardiac conduction through gap junctions and underpin functional electrical conduction [111]. Nevertheless, it is not clarified how the other leukocytes are useful for intracardiac homeostasis and conduction in a steady state. How the immune system is activated and operates systematically in AF requires further investigation.

### The role of gut microbes on the immune system

The community structure of the intestinal microbiota could dramatically determine the immune system. Low gene counts in the gut microbiota were correlated with high white blood cell counts [112]. The expression of PRRs such as TLRs in the intestine was also governed by intestinal bacteria, which facilitated host navigation between pathogens through PAMPs and commensal bacteria as well as the activation of immune sensory cells. *Prevotella* could coordinate the inflammatory response through TLR2 activation, leading to inflammation and T helper 17 cell immune response. Furthermore, the microbiota has established itself as an emerging mediator of macrophage function, which has a prominent contribution to the AF pathogenic mechanism (Fig. 3e). LPS-induced pro-inflammatory macrophages diminished atrial effective refractory period, elicited atrial electrical remodeling, and enhanced AF inducibility [88]. By directly inhibiting histone deacetylases 3, SCFA butyrate downregulated LPS-stimulated pro-inflammatory cytokines in mouse macrophages [113]. Together with SCFAs, indole derivatives of dietary and intestinal microbial origin also governed susceptibility to intestinal inflammation by activating the aryl hydrocarbon receptor pathway in macrophages [114]. The molecular basis of the microbiome in terms of its interface with the host’s innate and adaptive immune system is likewise being better elucidated.

### The gut-immune-heart axis in AF

Although the connection between intestinal flora, immunity, and the cardiovascular system was well established, direct evidence for the gut-immune-heart axis is scarce. One of them was the synthesis of TMAO by intestinal flora, which stimulated M1 macrophage polarization to exacerbate atrial structural remodeling and ultimately led to AF [55]. The expression of inflammatory markers owing to TMAO-activated thioredoxin-interacting protein-NLRP3 inflammasome could facilitate plaque formation by generating cholesterol-packed foamy macrophages in arteries [115]. TMAO also boosted protein kinase C/NF-κB activation and elevated expression of monocyte adhesion and vascular cell adhesion molecule 1 [116]. All these vascular pathological alterations might evolve into risk factors for AF. On the other side, transplantation with *Bacteroides dorei* and *Bacteroides vulgatus* or supplementation with *Lactobacillus plantarum* ATCC 14917 inhibited the formation of atherosclerotic lesions by downregulating serum oxidized low-density lipoprotein, IL-1β and TNF-α in the aorta [117, 118]. Anatomizing the intricate interactions between metabolic and immune systems will shed light on the biological basis of AF and how contemporary and future treatments may influence metabolism.

## Therapeutic potentials for modulating the microbiome

**Fig. 4:**
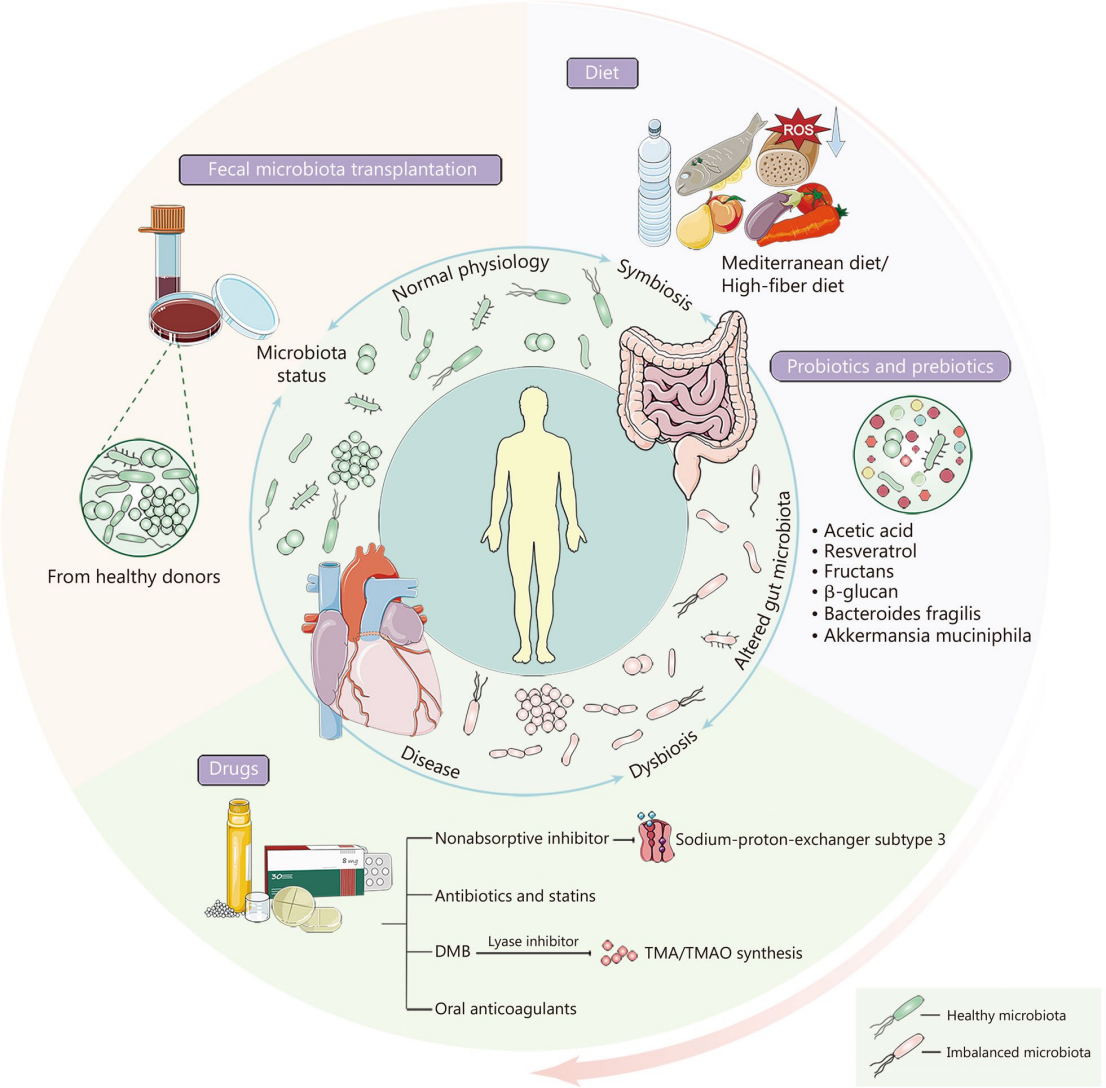
Candidates to formulate individualized measures for atrial fibrillation (AF) patients targeting microbes. The dysbiosis and symbiotic state of gut microbiota interact with the progression of diseases. The mechanistic link between the gut microbiome and AF poses these interactions as promising therapeutic targets. There are diverse interventions that can be implemented to prevent the deleterious biological effects of ecological dysbiosis, mainly including dietary interventions, probiotic/prebiotic supplementation, drugs, and fecal microbiota transplantation (FMT). Mediterranean diet and high-fiber diet can reduce circulating lipopolysaccharides (LPS) and trimethylamine oxide (TMAO) levels, alleviating oxidative stress and thus slowing AF development. The utilization of well-defined microbial components (probiotics) and non-microbial substances (prebiotics) that may modify the structure of the microbial community has revealed promising results in AF treatment. Moreover, nonabsorbable inhibitors, antibiotics, statins, and oral anticoagulants may exert effects on arrhythmic substrates through gut microbiota. 3,3-dimethyl-1-butanol (DMB), a prototype of lyase inhibitor, can alleviate AF progression by diminishing trimethylamine (TMA)/TMAO synthesis. FMT from healthy donors can alter the patients’ gut flora to treat the disease, but the therapeutic effect of FMT on AF remains to be further explored

Prospective intervention projects targeting intestinal flora in patients with AF are still absent. However, the above connections between altered intestinal flora components in AF patients, microbial metabolite generation, and AF risk proposed that intestinal flora might be an essential determinant of AF. Mechanistic linkages between gut microbiota and AF as well as meta-organismal routes that generated bioactive metabolites recognized by host receptors rendered these interactions promising therapeutic targets for modulating community output and host phenotype. Investigators have commenced the field of standardized and individualized microbiota-oriented therapeutic strategies to optimize clinical outcomes, primarily including dietary interventions, probiotic/prebiotic supplementation, drugs, and FMT (Fig. 4).

### Diet and probiotic/prebiotic in gut-heart axis

Currently, dietary modification is the foremost therapeutic method employed in clinical practice for the management of chronic metabolic diseases. Tabata et al. [36] demonstrated that the gut microbial composition of AF patients was affiliated with dietary habits. A high-fat diet resulted in disturbed intestinal flora and elevated circulating LPS levels in mice, predisposing them to AF [11]. Yet, the Mediterranean diet might be a plausible candidate for administering AF or its adverse events [119]. A prospective cohort study covering 690 AF patients revealed that adherence to the Mediterranean diet equated with higher glutathione peroxidase-3 antioxidant activity, which could mitigate oxidative stress to avert major adverse cardiovascular events [120]. Another single-center study including 912 AF patients suggested that LPS might advance AF by raising platelet activation and was negatively influenced by high compliance with the Mediterranean diet [41].

Alongside dietary patterns, specific dietary components reflecting cardiovascular disease risk and microbiota performed a valuable role. The TMAO pathway was a striking example [121], considering that Western diets were more abundant in nutrient precursors and that diets rich in carnitine and phosphatidylcholine were connected with elevated TMAO levels, while vegetarian or vegan diets lessened the nutrient precursors [122]. Of note, TMAO levels appeared to be governed more by the gut microbial composition than by dietary interventions, and remarkable discrepancies in TMAO generation were ascribed among individuals on a specific diet [123]. Furthermore, the extreme variation from animal-based to plant-based diets could shift the systemic and regional manufacture of SCFAs and thus be conducive to several proposed beneficial implications [20].

A few preclinical and clinical intervention investigations utilizing well-defined microbial components (probiotics) and non-microbial substances that might change the architecture of the microbial community (prebiotics) have exhibited promising outcomes. A recent example entailed a hypertension survey in rodents, where either a high-fiber diet or acetic acid alone declined blood pressure, cardiac fibrosis, and left ventricular hypertrophy [124]. Alimentary additives, including resveratrol, have been applied to thwart the pathogenesis of AF substrates among animal models [125]. Dietary inulin-based fructus supplementation has been proven to reverse carotid artery endothelial dysfunction by invigorating the nitric oxide synthase/nitric oxide pathway [126]. Β-glucan, a glucose polysaccharide producing SCFAs, could segregate cholesterol, scavenge ROS and exert immunostimulatory effects by mobilizing β-glucan receptors, including dectin-1 or CR3 on intestinal macrophages [127]. Supplementation with oat β-glucan raised high-density lipoprotein cholesterol and depressed plasma triglycerides and atherosclerosis, while simultaneously augmenting the genus *Akkermansia* in the intestinal tract [128]. Oral administration of *Bacteroides fragilis* could considerably subdue the inflammatory response via augmenting Treg cells in D-galactose-induced aging rats, thereby prohibiting atrial structural remodeling and restraining AF boosting [129]. Nevertheless, the magnitude of these researches tended to be relatively modest, and the adaptation in clinical practice awaited deeper scrutiny. Moreover, it was recently demonstrated by Luo et al. [55] that oral supplementation of *Akkermansia muciniphila* in rats ameliorated pro-AF properties which were induced by cold exposure. This probiotic has garnered interest for its application in human clinical trials [130]. Nonetheless, an aspect of the dilemma in probiotic research was that secondary impacts such as host immune education and function on the microbial community might contribute to the deleterious effects if the tremendous scale of gut microbial community and inter-individual heterogeneity in the community structure was observed.

### Drug-gut microbiota interactions

Adjustment of the gastrointestinal luminal environment by selective pharmacological interruption with the absorption of dietary glucose, ions, proteins, or fat might pose an alternative pathway. For instance, suppression of intestinal sodium-proton-exchanger subtype 3 with the oral nonabsorptive inhibitor could abrogate the progression of arrhythmogenic substrates for AF in hypertensive rats [131]. Additionally, oral antibiotics and statins could reduce or enhance the growth velocity of intestinal flora and interfere with microbial metabolite generation. Despite the multiple surveys reporting the relationships between atherosclerotic plaques and pathogens, numerous prospective randomized antibiotic trials have so far failed to demonstrate any clinical benefit [132]. Considering that recolonization of the microbial community after withdrawal of antibiotic treatment was difficult to predict, antibiotic administrations might seem more amenable to rooting out the true pathogen rather than as a long-term prophylactic measure.

There was a bidirectional effect of oral anticoagulants (OACs) on the gut flora of AF patients. OACs were the cornerstone of AF management, which could reduce stroke and death risk. A previous study revealed that gut microbiota could influence the bioavailability and efficacy of OACs [133]. Intestinal flora might have an indirect effect on OACs through the metabolites generated, or a direct effect on vitamin K-producing bacteria and structural modifications of drug molecules. Correspondingly, Li et al. [134] demonstrated that the implementation of OACs in AF patients might mitigate inflammation, atherosclerosis, and thrombosis by altering the abundance of gut microbiota. Nonetheless, OACs might raise the risk of bleeding in the gastrointestinal tract and other parts of the body through certain potential pathogens. There were no available studies exploring whether exogenous supplementation of OACs-reduced microbiota could lower the risk of bleeding events.

Due to the identification of microbial enzymes that synergistically converted nutrients such as choline or carnitine to TMA, several small molecule inhibitors reversing adverse cardiovascular effects have been identified in experimental studies [135, 136]. The prototype of a lyase inhibitor was DMB, which could inhibit TMA/TMAO synthesis in vivo mouse models and in microbial cell culture without compromising the survival of microbial cells. Mice treated with DMB experienced diminished atherosclerotic lesions, attenuated foam cell formation, and alleviated cardiovascular disease progression [137]. More importantly, in experimental animal models, DMB was demonstrated to mitigate the development of cold and diabetes-related AF by curtailing the synthesis of TMAO [55, 72]. Even though human clinical investigations of choline TMA lyase blockers have not been investigated, there are substantial endeavors underway in this domain.

As AF patients might receive antiarrhythmic drugs or anticoagulants during long-term treatment, examining the interaction between gut flora and drugs was of extensive interest. There was a vast diversity in the metabolism of drugs by gut microbes, which might entail potential alterations in responsiveness. The divergent drug-microbe interfaces between different individuals are another area that deserves deeper study and displays prospects for practical application in personalized medicine and drug exploration efforts.

### FMT

While most frequently utilized for mechanistic purposes, FMT has gained prominence as a regimen for well-defined indications such as *Clostridium difficile* colitis. Investigational employment of FMT from human or mouse origin, transplanted to germ-free mice that were exclusively deprived of the microbiome, has immensely expanded the comprehension of gut microbiome’s causal roles in registering and contributing to cardiac arrhythmia. Several researches using FMT rat models offered original causes of gut microbiota dysbiosis for the onset of age, obesity, and cold-associated AF [10, 11, 55]. Furthermore, Zhang et al. [10] also demonstrated that aged rats colonized with young microbiota rehabilitated atrial NLRP3-inflammasome activity and gut structure, thereby curtailing the pathogenesis of age-related AF. Nevertheless, the limited human data and the mechanistic explanation of this effect necessitate further studies. In a small randomized double-blind pilot trial, single vegan-donor FMT altered the composition of the gut microbiota in subjects with metabolic syndrome but an inability to influence TMAO manufacture or atherogenic pro-inflammatory status [138]. Both the short-term follow-up period and the small sample size might account for the lack of discernible effects. In addition, Manrique et al. [139] uncovered that FMT from healthy donors remarkably altered the gut bacteriophage community in patients with metabolic syndrome. A clearer comprehension of causative versus “passenger” bacteria, as well as the participation of non-bacterial ingredients such as virome and fungome, might assist in specifying a clear microbiological profile that maximized efficacy while simultaneously addressing the security of the procedure and its prolonged impact on the recipients.

## Conclusions and future perspectives

Whilst our perception about how microbiome influences AF is still nascent, the speed with which novel revelations are arising is striking. Several preclinical surveys have explicitly proposed that intestinal flora could potentiate the development and maintenance of AF under experimental conditions, but the underlying mechanisms remain unarticulated. Furthermore, it is uncertain whether these discoveries can be interpreted in the clinical context and whether modifications in intestinal flora may pose new therapeutic aspects for AF administration. There is a demand for well-designed prospective interventional studies to verify the causal relationship between AF and gut microbiome and to appraise whether aberrant gut microbiome is only a marker for other AF risk factors or an isolated indicator. It is instructive to explore whether the characteristics of gut microbiome under different pathophysiological conditions and its derived metabolites can be exploited as biomarkers of prognosis or the curative performance of ablation therapy in AF patients.

The oral cavity is also colonized with a slightly heterogeneous microbial society, and ever-increasing proportions of oral bacteria have been correlated with cardiovascular disease [140]. Oral diseases and cardiovascular diseases share substantial overlapping risk factors as well as joint biomarkers and genetic susceptibility variants, signifying an underlying shared pathophysiology of periodontitis and cardiovascular diseases [141]. Prior observational investigation has upheld the connection between periodontitis and AF irrespective of known confounders [142]. In an animal model, periodontitis has been observed to strengthen the immune activation of the atrial myocardium, thereby subverting the electrophysiological properties of the atria [143]. A recent population-based cohort study of 161,286 subjects revealed that improvements in oral hygiene such as regular professional dental cleanings and frequent tooth brushing were linked to a decreased risk of AF [144]. However, few experiments have been conducted to study the role and mechanisms of oral flora in AF pathophysiology. Future investigations may benefit from interrogating the connection between the characteristics as well as the metabolism of oral flora and AF. Considering the treatment of periodontitis as a highly feasible and daily manageable measure for AF prevention, randomized clinical trials dealing with standardized oral flora interventions are warranted to demonstrate the advantage of periodontal treatment in mitigating AF risk.

Because of the heterogeneity and transparency of the gut microbiome as well as state-of-the-art technological advances, meta-omics approaches hold a prospect as a gateway to unveiling host-microbiome interactions and evaluating causality in the clinical milieu. Nevertheless, it has restrictions and requires further innovation. A portion of the data could not be allotted on account of insufficient reference databases, especially the scarcity of eukaryotic and viral matches, which hampered the extraction of organismal and functional information. Currently, only the bacterial community has been extensively studied, whilst other constituents of the gut microbiota, such as viruses, fungi, or archaea, have not been prolifically explored. Zuo et al. [38] have demonstrated that enterovirus features were correlated with AF for the first time, which has tremendous potential value in anticipating ablation outcomes (Table 1). Therefore, the conjunction of these members in microbial-AF association analysis may unlock a fresh door for potential therapeutic approaches. Furthermore, another difficulty of special pertinence to microbiome studies is the drastic inter-individual variation in the configuration of the gut microbiome and the subsequent host metabolism induced by dietary interventions. In turn, applying machine learning to datasets encompassing diet, gut flora, and genetics might deliver the solution. Apart from direct causal routes, specific bacteria or microbiota constituents may be implied by incident or simply as modifying variables in drug regimens and genetic studies. Thus, the microbiome remains a crucial segment of personalized and precision medicine.

## Data Availability

Not applicable.

## Abbreviations

AF: Atrial fibrillation
ANS: Autonomic nervous system
BAs: Bile acids
CaMKII: Calmodulin kinase II
CASP1: Caspase-1
CFs: Cardiac fibroblasts
CMs: Cardiac myocytes
Cx: Connexin
DAMPs: Damage-associated molecular patterns
DMB: Dimethylbutanol
FMT: Fecal microbiota transplantation
GPR43: G protein-coupled receptor 43
IL: Interleukin
LPS: Lipopolysaccharides
miRNAs or miRs: MicroRNAs
NF-κB: Nuclear factor-κB
NLRP3: NACHT, LRR, and PYD domains-containing protein-3
OACs: Oral anticoagulants
PAGln: Phenylacetylglutamine
PAMPs: Pathogen-associated molecular patterns
PRRs: Pattern recognition receptors
ROS: Reactive oxygen species
RyR2: Ryanodine receptor 2
SCFAs: Short-chain fatty acids
TGF-β: Transforming growth factor-β
TMA: Trimethylamine
TMAO: Trimethylamine oxide
TNF: Tumor necrosis factor
TLR: Toll-like receptor

## References

[1] Kim JE, Li B, Fei L, Horne R, Lee D, Loe AK (2022). Gut microbiota promotes stem cell differentiation through macrophage and mesenchymal niches in early postnatal development. Immunity.

[2] Xiao W, Su J, Gao X, Yang H, Weng R, Ni W (2022). The microbiota-gut-brain axis participates in chronic cerebral hypoperfusion by disrupting the metabolism of short-chain fatty acids. Microbiome.

[3] Manor O, Dai CL, Kornilov SA, Smith B, Price ND, Lovejoy JC (2020). Health and disease markers correlate with gut microbiome composition across thousands of people. Nat Commun.

[4] Cui GY, Rao BC, Zeng ZH, Wang XM, Ren T, Wang HY (2022). Characterization of oral and gut microbiome and plasma metabolomics in COVID-19 patients after 1-year follow-up. Mil Med Res.

[5] Rao BC, Zhang GZ, Zou YW, Ren T, Ren HY, Liu C (2022). Alterations in the human oral microbiome in cholangiocarcinoma. Mil Med Res.

[6] Li LY, Han J, Wu L, Fang C, Li WG, Gu JM (2022). Alterations of gut microbiota diversity, composition and metabonomics in testosterone-induced benign prostatic hyperplasia rats. Mil Med Res.

[7] Patti G, Lucerna M, Pecen L, Siller-Matula JM, Cavallari I, Kirchhof P (2017). Thromboembolic risk, bleeding outcomes and effect of different antithrombotic strategies in very elderly patients with atrial fibrillation: a sub-analysis from the PREFER in AF (PREvention oF thromboembolic events-European Registry in Atrial Fibrillation). J Am Heart Assoc.

[8] Ngo L, Woodman R, Denman R, Walters TE, Yang IA, Ranasinghe I (2023). Longitudinal risk of death, hospitalizations for atrial fibrillation, and cardiovascular events following catheter ablation of atrial fibrillation: a cohort study. Eur Heart J Qual Care Clin Outcomes.

[9] Wang M, Xiong H, Lu L, Zhu T, Jiang H (2022). Serum lipopolysaccharide is associated with the recurrence of atrial fibrillation after radiofrequency ablation by increasing systemic inflammation and atrial fibrosis. Oxid Med Cell Longev.

[10] Zhang Y, Zhang S, Li B, Luo Y, Gong Y, Jin X (2022). Gut microbiota dysbiosis promotes age-related atrial fibrillation by lipopolysaccharide and glucose-induced activation of NLRP3-inflammasome. Cardiovasc Res.

[11] Kong B, Fu H, Xiao Z, Zhou Y, Shuai W, Huang H (2022). Gut microbiota dysbiosis induced by a high-fat diet increases susceptibility to atrial fibrillation. Can J Cardiol.

[12] Drapkina OM, Yafarova AA, Kaburova AN, Kiselev AR (2022). Targeting gut microbiota as a novel strategy for prevention and treatment of hypertension, atrial fibrillation and heart failure: current knowledge and future perspectives. Biomedicines.

[13] Gawalko M, Agbaedeng TA, Saljic A, Muller DN, Wilck N, Schnabel R (2022). Gut microbiota, dysbiosis and atrial fibrillation. Arrhythmogenic mechanisms and potential clinical implications. Cardiovasc Res.

[14] Lu D, Zou X, Zhang H (2022). The relationship between atrial fibrillation and intestinal flora with its metabolites. Front Cardiovasc Med.

[15] Lagier JC, Khelaifia S, Alou MT, Ndongo S, Dione N, Hugon P (2016). Culture of previously uncultured members of the human gut microbiota by culturomics. Nat Microbiol.

[16] Pasolli E, Asnicar F, Manara S, Zolfo M, Karcher N, Armanini F (2019). Extensive unexplored human microbiome diversity revealed by over 150,000 genomes from metagenomes spanning age, geography, and lifestyle. Cell.

[17] Abubucker S, Segata N, Goll J, Schubert AM, Izard J, Cantarel BL (2012). Metabolic reconstruction for metagenomic data and its application to the human microbiome. PLoS Comput Biol.

[18] Cambiaghi A, Ferrario M, Masseroli M (2017). Analysis of metabolomic data: tools, current strategies and future challenges for omics data integration. Brief Bioinform.

[19] Nicholson JK, Holmes E, Kinross J, Burcelin R, Gibson G, Jia W (2012). Host-gut microbiota metabolic interactions. Science.

[20] David LA, Maurice CF, Carmody RN, Gootenberg DB, Button JE, Wolfe BE (2014). Diet rapidly and reproducibly alters the human gut microbiome. Nature.

[21] Bikel S, Valdez-Lara A, Cornejo-Granados F, Rico K, Canizales-Quinteros S, Soberón X (2015). Combining metagenomics, metatranscriptomics and viromics to explore novel microbial interactions: towards a systems-level understanding of human microbiome. Comput Struct Biotechnol J.

[22] Zhang X, Figeys D (2019). Perspective and guidelines for metaproteomics in microbiome studies. J Proteome Res.

[23] Ali RO, Quinn GM, Umarova R, Haddad JA, Zhang GY, Townsend EC (2023). Longitudinal multi-omics analyses of the gut-liver axis reveals metabolic dysregulation in hepatitis C infection and cirrhosis. Nat Microbiol.

[24] Mills RH, Dulai PS, Vazquez-Baeza Y, Sauceda C, Daniel N, Gerner RR (2022). Multi-omics analyses of the ulcerative colitis gut microbiome link Bacteroides vulgatus proteases with disease severity. Nat Microbiol.

[25] Blanco-Miguez A, Fdez-Riverola F, Sanchez B, Lourenco A (2019). Resources and tools for the high-throughput, multi-omic study of intestinal microbiota. Brief Bioinform.

[26] Weiss S, Van Treuren W, Lozupone C, Faust K, Friedman J, Deng Y (2016). Correlation detection strategies in microbial data sets vary widely in sensitivity and precision. ISME J.

[27] Chaudhary K, Poirion OB, Lu L, Garmire LX (2018). Deep learning-based multi-omics integration robustly predicts survival in liver cancer. Clin Cancer Res.

[28] Zuo K, Li J, Li K, Hu C, Gao Y, Chen M (2019). Disordered gut microbiota and alterations in metabolic patterns are associated with atrial fibrillation. Gigascience..

[29] Zuo K, Li J, Wang P, Liu Y, Liu Z, Yin X (2019). Duration of persistent atrial fibrillation is associated with alterations in human gut microbiota and metabolic phenotypes. mSystems.

[30] Zuo K, Yin X, Li K, Zhang J, Wang P, Jiao J (2020). Different types of atrial fibrillation share patterns of gut microbiota dysbiosis. mSphere.

[31] Li J, Zuo K, Zhang J, Hu C, Wang P, Jiao J (2020). Shifts in gut microbiome and metabolome are associated with risk of recurrent atrial fibrillation. J Cell Mol Med.

[32] Xu F, Fu Y, Sun TY, Jiang Z, Miao Z, Shuai M (2020). The interplay between host genetics and the gut microbiome reveals common and distinct microbiome features for complex human diseases. Microbiome.

[33] Huang K, Wang Y, Bai Y, Luo Q, Lin X, Yang Q (2022). Gut microbiota and metabolites in atrial fibrillation patients and their changes after catheter ablation. Microbiol Spectr.

[34] Palmu J, Borschel CS, Ortega-Alonso A, Marko L, Inouye M, Jousilahti P (2023). Gut microbiome and atrial fibrillation-results from a large population-based study. EBioMedicine.

[35] Wang Y, He Y, Li R, Jiang H, Tao D, Zhao K (2023). Gut microbiota in patients with postoperative atrial fibrillation undergoing off-pump coronary bypass graft surgery. J Clin Med.

[36] Tabata T, Yamashita T, Hosomi K, Park J, Hayashi T, Yoshida N (2021). Gut microbial composition in patients with atrial fibrillation: effects of diet and drugs. Heart Vessels.

[37] Fang C, Zuo K, Zhang W, Zhong J, Li J, Xu L (2022). Association between gut microbiota dysbiosis and the CHA2DS2-VASc score in atrial fibrillation patients. Int J Clin Pract.

[38] Zuo K, Li J, Fang C, Zhong J, Xu L, Yang X (2022). Alterations of gut viral signals in atrial fibrillation: complex linkage with gut bacteriome. Aging.

[39] Imhann F, Bonder MJ, Vich Vila A, Fu J, Mujagic Z, Vork L (2016). Proton pump inhibitors affect the gut microbiome. Gut.

[40] Zuo K, Liu X, Wang P, Jiao J, Han C, Liu Z (2020). Metagenomic data-mining reveals enrichment of trimethylamine-N-oxide synthesis in gut microbiome in atrial fibrillation patients. BMC Genomics.

[41] Pastori D, Carnevale R, Nocella C, Novo M, Santulli M, Cammisotto V (2017). Gut-derived serum lipopolysaccharide is associated with enhanced risk of major adverse cardiovascular events in atrial fibrillation: effect of adherence to Mediterranean diet. J Am Heart Assoc.

[42] Gong D, Zhang L, Zhang Y, Wang F, Zhao Z, Zhou X (2019). Gut microbial metabolite trimethylamine N-oxide is related to thrombus formation in atrial fibrillation patients. Am J Med Sci.

[43] Luciani M, Muller D, Vanetta C, Diteepeng T, von Eckardstein A, Aeschbacher S (2023). Trimethylamine-N-oxide is associated with cardiovascular mortality and vascular brain lesions in patients with atrial fibrillation. Heart.

[44] Papandreou C, Bullo M, Hernandez-Alonso P, Ruiz-Canela M, Li J, Guasch-Ferre M (2021). Choline metabolism and risk of atrial fibrillation and heart failure in the PREDIMED study. Clin Chem.

[45] Jia J, Dou P, Gao M, Kong X, Li C, Liu Z (2019). Assessment of causal direction between gut microbiota-dependent metabolites and cardiometabolic health: a bidirectional mendelian randomization analysis. Diabetes.

[46] Zhang J, Zuo K, Fang C, Yin X, Liu X, Zhong J (2021). Altered synthesis of genes associated with short-chain fatty acids in the gut of patients with atrial fibrillation. BMC Genomics.

[47] Zuo K, Fang C, Liu Z, Fu Y, Liu Y, Liu L (2022). Commensal microbe-derived SCFA alleviates atrial fibrillation via GPR43/NLRP3 signaling. Int J Biol Sci.

[48] Fan PC, Chang JC, Lin CN, Lee CC, Chen YT, Chu PH (2019). Serum indoxyl sulfate predicts adverse cardiovascular events in patients with chronic kidney disease. J Formos Med Assoc.

[49] Alonso A, Yu B, Sun YV, Chen LY, Loehr LR, O'Neal WT (2019). Serum metabolomics and incidence of atrial fibrillation (from the atherosclerosis risk in communities study). Am J Cardiol.

[50] Zuo K, Fang C, Gao Y, Fu Y, Wang H, Li J (2023). Suppression of the gut microbiota-bile acid-FGF19 axis in patients with atrial fibrillation. Cell Prolif.

[51] Inzaugarat ME, Johnson CD, Holtmann TM, McGeough MD, Trautwein C, Papouchado BG (2019). NLR family pyrin domain-containing 3 inflammasome activation in hepatic stellate cells induces liver fibrosis in mice. Hepatology.

[52] Frissen M, Liao L, Schneider KM, Djudjaj S, Haybaeck J, Wree A (2021). Bidirectional role of NLRP3 during acute and chronic cholestatic liver injury. Hepatology.

[53] Caceres FT, Gaspari TA, Samuel CS, Pinar AA (2019). Serelaxin inhibits the profibrotic TGF-beta1/IL-1beta axis by targeting TLR-4 and the NLRP3 inflammasome in cardiac myofibroblasts. FASEB J.

[54] Yao C, Veleva T, Scott L, Cao S, Li L, Chen G (2018). Enhanced cardiomyocyte NLRP3 inflammasome signaling promotes atrial fibrillation. Circulation.

[55] Luo Y, Zhang Y, Han X, Yuan Y, Zhou Y, Gao Y (2022). Akkermansia muciniphila prevents cold-related atrial fibrillation in rats by modulation of TMAO induced cardiac pyroptosis. EBioMedicine.

[56] Yang W, Zhang S, Zhu J, Jiang H, Jia D, Ou T (2019). Gut microbe-derived metabolite trimethylamine N-oxide accelerates fibroblast-myofibroblast differentiation and induces cardiac fibrosis. J Mol Cell Cardiol.

[57] Yue L, Xie J, Nattel S (2011). Molecular determinants of cardiac fibroblast electrical function and therapeutic implications for atrial fibrillation. Cardiovasc Res.

[58] Shi J, Zhao Y, Wang K, Shi X, Wang Y, Huang H (2015). Cleavage of GSDMD by inflammatory caspases determines pyroptotic cell death. Nature.

[59] Bartolomaeus H, Balogh A, Yakoub M, Homann S, Marko L, Hoges S (2019). Short-chain fatty acid propionate protects from hypertensive cardiovascular damage. Circulation.

[60] Fang C, Zuo K, Jiao K, Zhu X, Fu Y, Zhong J (2022). PAGln, an atrial fibrillation-linked gut microbial metabolite, acts as a promoter of atrial myocyte injury. Biomolecules.

[61] Nemet I, Saha PP, Gupta N, Zhu W, Romano KA, Skye SM (2020). A cardiovascular disease-linked gut microbial metabolite acts via adrenergic receptors. Cell.

[62] Kilts JD, Gerhardt MA, Richardson MD, Sreeram G, Mackensen GB, Grocott HP (2000). Beta(2)-adrenergic and several other G protein-coupled receptors in human atrial membranes activate both G(s) and G(i). Circ Res.

[63] Trappe K, Thomas D, Bikou O, Kelemen K, Lugenbiel P, Voss F (2013). Suppression of persistent atrial fibrillation by genetic knockdown of caspase 3: a pre-clinical pilot study. Eur Heart J.

[64] Yue L, Feng J, Gaspo R, Li GR, Wang Z, Nattel S (1997). Ionic remodeling underlying action potential changes in a canine model of atrial fibrillation. Circ Res.

[65] Cha TJ, Ehrlich JR, Chartier D, Qi XY, Xiao L, Nattel S (2006). Kir3-based inward rectifier potassium current: potential role in atrial tachycardia remodeling effects on atrial repolarization and arrhythmias. Circulation.

[66] Igarashi T, Finet JE, Takeuchi A, Fujino Y, Strom M, Greener ID (2012). Connexin gene transfer preserves conduction velocity and prevents atrial fibrillation. Circulation.

[67] Fang C, Zuo K, Liu Z, Liu Y, Liu L, Wang Y (2023). Disordered gut microbiota promotes atrial fibrillation by aggravated conduction disturbance and unbalanced linoleic acid/SIRT1 signaling. Biochem Pharmacol.

[68] Rainer PP, Primessnig U, Harenkamp S, Doleschal B, Wallner M, Fauler G (2013). Bile acids induce arrhythmias in human atrial myocardium–implications for altered serum bile acid composition in patients with atrial fibrillation. Heart.

[69] Sheikh Abdul Kadir SH, Miragoli M, Abu-Hayyeh S, Moshkov AV, Xie Q, Keitel V (2010). Bile acid-induced arrhythmia is mediated by muscarinic M2 receptors in neonatal rat cardiomyocytes. PLoS ONE.

[70] Hao H, Cao L, Jiang C, Che Y, Zhang S, Takahashi S (2017). Farnesoid X receptor regulation of the NLRP3 inflammasome underlies cholestasis-associated sepsis. Cell Metab.

[71] Wiegerinck RF, van Veen TAB, Belterman CN, Schumacher CA, Noorman M, de Bakker JMT (2008). Transmural dispersion of refractoriness and conduction velocity is associated with heterogeneously reduced connexin43 in a rabbit model of heart failure. Heart Rhythm.

[72] Jiang WY, Huo JY, Wang SC, Cheng YD, Lyu YT, Jiang ZX (2022). Trimethylamine N-oxide facilitates the progression of atrial fibrillation in rats with type 2 diabetes by aggravating cardiac inflammation and connexin remodeling. J Physiol Biochem.

[73] Zhang L, Xie F, Tang H, Zhang X, Hu J, Zhong X (2022). Gut microbial metabolite TMAO increases peritoneal inflammation and peritonitis risk in peritoneal dialysis patients. Transl Res.

[74] Lazzerini PE, Laghi-Pasini F, Acampa M, Srivastava U, Bertolozzi I, Giabbani B (2019). Systemic inflammation rapidly induces reversible atrial electrical remodeling: the role of interleukin-6-mediated changes in connexin expression. J Am Heart Assoc.

[75] Ryu K, Li L, Khrestian CM, Matsumoto N, Sahadevan J, Ruehr ML (2007). Effects of sterile pericarditis on connexins 40 and 43 in the atria: correlation with abnormal conduction and atrial arrhythmias. Am J Physiol Heart Circ Physiol.

[76] Sawaya SE, Rajawat YS, Rami TG, Szalai G, Price RL, Sivasubramanian N (2007). Downregulation of connexin40 and increased prevalence of atrial arrhythmias in transgenic mice with cardiac-restricted overexpression of tumor necrosis factor. Am J Physiol Heart Circ Physiol.

[77] Chen ML, Zhu XH, Ran L, Lang HD, Yi L, Mi MT (2017). Trimethylamine-N-oxide induces vascular inflammation by activating the NLRP3 inflammasome through the SIRT3-SOD2-mtROS signaling pathway. J Am Heart Assoc.

[78] Hou Y, Scherlag BJ, Lin J, Zhang Y, Lu Z, Truong K (2007). Ganglionated plexi modulate extrinsic cardiac autonomic nerve input: effects on sinus rate, atrioventricular conduction, refractoriness, and inducibility of atrial fibrillation. J Am Coll Cardiol.

[79] Nishida K, Maguy A, Sakabe M, Comtois P, Inoue H, Nattel S (2011). The role of pulmonary veins vs. autonomic ganglia in different experimental substrates of canine atrial fibrillation. Cardiovasc Res.

[80] Yu L, Meng G, Huang B, Zhou X, Stavrakis S, Wang M (2018). A potential relationship between gut microbes and atrial fibrillation: trimethylamine N-oxide, a gut microbe-derived metabolite, facilitates the progression of atrial fibrillation. Int J Cardiol.

[81] Seldin MM, Meng Y, Qi H, Zhu W, Wang Z, Hazen SL (2016). Trimethylamine N-oxide promotes vascular inflammation through signaling of mitogen-activated protein kinase and nuclear factor-kappaB. J Am Heart Assoc.

[82] Wei SG, Yu Y, Zhang ZH, Felder RB (2015). Proinflammatory cytokines upregulate sympathoexcitatory mechanisms in the subfornical organ of the rat. Hypertension.

[83] Bravo JA, Forsythe P, Chew MV, Escaravage E, Savignac HM, Dinan TG (2011). Ingestion of Lactobacillus strain regulates emotional behavior and central GABA receptor expression in a mouse via the vagus nerve. Proc Natl Acad Sci U S A.

[84] Kimura I, Inoue D, Maeda T, Hara T, Ichimura A, Miyauchi S (2011). Short-chain fatty acids and ketones directly regulate sympathetic nervous system via G protein-coupled receptor 41 (GPR41). Proc Natl Acad Sci U S A.

[85] Goehler LE, Park SM, Opitz N, Lyte M, Gaykema RP (2008). Campylobacter jejuni infection increases anxiety-like behavior in the holeboard: possible anatomical substrates for viscerosensory modulation of exploratory behavior. Brain Behav Immun.

[86] Qiu M, Huang K, Liu Y, Yang Y, Tang H, Liu X (2019). Modulation of intestinal microbiota by glycyrrhizic acid prevents high-fat diet-enhanced pre-metastatic niche formation and metastasis. Mucosal Immunol.

[87] Zhang XY, Chen J, Yi K, Peng L, Xie J, Gou X (2020). Phlorizin ameliorates obesity-associated endotoxemia and insulin resistance in high-fat diet-fed mice by targeting the gut microbiota and intestinal barrier integrity. Gut Microbes.

[88] Sun Z, Zhou D, Xie X, Wang S, Wang Z, Zhao W (2016). Cross-talk between macrophages and atrial myocytes in atrial fibrillation. Basic Res Cardiol.

[89] Chen WT, Chen YC, Hsieh MH, Huang SY, Kao YH, Chen YA (2015). The uremic toxin indoxyl sulfate increases pulmonary vein and atrial arrhythmogenesis. J Cardiovasc Electrophysiol.

[90] Li D, Shinagawa K, Pang L, Leung TK, Cardin S, Wang Z (2001). Effects of angiotensin-converting enzyme inhibition on the development of the atrial fibrillation substrate in dogs with ventricular tachypacing-induced congestive heart failure. Circulation.

[91] Qiu H, Ji C, Liu W, Wu Y, Lu Z, Lin Q (2018). Chronic kidney disease increases atrial fibrillation inducibility: involvement of inflammation, atrial fibrosis, and connexins. Front Physiol.

[92] Qiu H, Liu W, Lan T, Pan W, Chen X, Wu H (2018). Salvianolate reduces atrial fibrillation through suppressing atrial interstitial fibrosis by inhibiting TGF-beta1/Smad2/3 and TXNIP/NLRP3 inflammasome signaling pathways in post-MI rats. Phytomedicine.

[93] Fang J, Kong B, Shuai W, Xiao Z, Dai C, Qin T (2021). Ferroportin-mediated ferroptosis involved in new-onset atrial fibrillation with LPS-induced endotoxemia. Eur J Pharmacol.

[94] Liu D, Yang M, Yao Y, He S, Wang Y, Cao Z (2022). Cardiac fibroblasts promote ferroptosis in atrial fibrillation by secreting exo-miR-23a-3p targeting SLC7A11. Oxid Med Cell Longev.

[95] Li YD, Hong YF, Yusufuaji Y, Tang BP, Zhou XH, Xu GJ (2015). Altered expression of hyperpolarization-activated cyclic nucleotide-gated channels and microRNA-1 and -133 in patients with age-associated atrial fibrillation. Mol Med Rep.

[96] Zhao L, Zhou T, Chen J, Cai W, Shi R, Duan Y (2022). Colon specific delivery of miR-155 inhibitor alleviates estrogen deficiency related phenotype via microbiota remodeling. Drug Deliv.

[97] Vikram A, Kim YR, Kumar S, Li Q, Kassan M, Jacobs JS (2016). Vascular microRNA-204 is remotely governed by the microbiome and impairs endothelium-dependent vasorelaxation by downregulating Sirtuin1. Nat Commun.

[98] Huxley RR, Lopez FL, Folsom AR, Agarwal SK, Loehr LR, Soliman EZ (2011). Absolute and attributable risks of atrial fibrillation in relation to optimal and borderline risk factors: the Atherosclerosis Risk in Communities (ARIC) study. Circulation.

[99] Vinter N, Christesen AMS, Mortensen LS, Urbonaviciene G, Lindholt J, Johnsen SP (2018). Coronary artery calcium score and the long-term risk of atrial fibrillation in patients undergoing non-contrast cardiac computed tomography for suspected coronary artery disease: a Danish registry-based cohort study. Eur Heart J Cardiovasc Imaging.

[100] Mountantonakis SE, Grau-Sepulveda MV, Bhatt DL, Hernandez AF, Peterson ED, Fonarow GC (2012). Presence of atrial fibrillation is independently associated with adverse outcomes in patients hospitalized with heart failure: an analysis of get with the guidelines-heart failure. Circ Heart Fail.

[101] Heidt T, Courties G, Dutta P, Sager HB, Sebas M, Iwamoto Y (2014). Differential contribution of monocytes to heart macrophages in steady-state and after myocardial infarction. Circ Res.

[102] Chen MC, Chang JP, Liu WH, Yang CH, Chen YL, Tsai TH (2008). Increased inflammatory cell infiltration in the atrial myocardium of patients with atrial fibrillation. Am J Cardiol.

[103] Monnerat G, Alarcon ML, Vasconcellos LR, Hochman-Mendez C, Brasil G, Bassani RA (2016). Macrophage-dependent IL-1beta production induces cardiac arrhythmias in diabetic mice. Nat Commun.

[104] Kuwahara F, Kai H, Tokuda K, Takeya M, Takeshita A, Egashira K (2004). Hypertensive myocardial fibrosis and diastolic dysfunction: another model of inflammation?. Hypertension.

[105] Lindsey ML, Escobar GP, Mukherjee R, Goshorn DK, Sheats NJ, Bruce JA (2006). Matrix metalloproteinase-7 affects connexin-43 levels, electrical conduction, and survival after myocardial infarction. Circulation.

[106] Yang Y, Ma L, Wang C, Song M, Li C, Chen M (2020). Matrix metalloproteinase-7 in platelet-activated macrophages accounts for cardiac remodeling in uremic mice. Basic Res Cardiol.

[107] Kao YH, Chen YC, Cheng CC, Lee TI, Chen YJ, Chen SA (2010). Tumor necrosis factor-alpha decreases sarcoplasmic reticulum Ca^2+^-ATPase expressions via the promoter methylation in cardiomyocytes. Crit Care Med.

[108] Cacheux M, Strauss B, Raad N, Ilkan Z, Hu J, Benard L (2019). Cardiomyocyte-specific STIM1 (stromal interaction molecule 1) depletion in the adult heart promotes the development of arrhythmogenic discordant alternans. Circ Arrhythm Electrophysiol.

[109] Nicolas-Avila JA, Lechuga-Vieco AV, Esteban-Martinez L, Sanchez-Diaz M, Diaz-Garcia E, Santiago DJ (2020). A network of macrophages supports mitochondrial homeostasis in the heart. Cell.

[110] Hulsmans M, Clauss S, Xiao L, Aguirre AD, King KR, Hanley A (2017). Macrophages facilitate electrical conduction in the heart. Cell.

[111] De Jesus NM, Wang L, Herren AW, Wang J, Shenasa F, Bers DM (2015). Atherosclerosis exacerbates arrhythmia following myocardial infarction: role of myocardial inflammation. Heart Rhythm.

[112] Le Chatelier E, Nielsen T, Qin J, Prifti E, Hildebrand F, Falony G (2013). Richness of human gut microbiome correlates with metabolic markers. Nature.

[113] Schulthess J, Pandey S, Capitani M, Rue-Albrecht KC, Arnold I, Franchini F (2019). The short chain fatty acid butyrate imprints an antimicrobial program in macrophages. Immunity.

[114] Chng SH, Kundu P, Dominguez-Brauer C, Teo WL, Kawajiri K, Fujii-Kuriyama Y, et al. Ablating the aryl hydrocarbon receptor (AhR) in CD11c^+^ cells perturbs intestinal epithelium development and intestinal immunity. Sci Rep. 2016;6:23820. 10.1038/srep23820 PMC4828637 27068235

[115] Sun X, Jiao X, Ma Y, Liu Y, Zhang L, He Y (2016). Trimethylamine N-oxide induces inflammation and endothelial dysfunction in human umbilical vein endothelial cells via activating ROS-TXNIP-NLRP3 inflammasome. Biochem Biophys Res Commun.

[116] Ma G, Pan B, Chen Y, Guo C, Zhao M, Zheng L (2017). Trimethylamine N-oxide in atherogenesis: impairing endothelial self-repair capacity and enhancing monocyte adhesion. Biosci Rep.

[117] Yoshida N, Emoto T, Yamashita T, Watanabe H, Hayashi T, Tabata T (2018). Bacteroides vulgatus and Bacteroides dorei reduce gut microbial lipopolysaccharide production and inhibit atherosclerosis. Circulation.

[118] Hassan A, Din AU, Zhu Y, Zhang K, Li T, Wang Y (2020). Anti-atherosclerotic effects of Lactobacillus plantarum ATCC 14917 in ApoE(-/-) mice through modulation of proinflammatory cytokines and oxidative stress. Appl Microbiol Biotechnol.

[119] Barrio-Lopez MT, Ruiz-Canela M, Ramos P, Tercedor L, Ibañez Criado JL, Ortiz M (2020). PREvention of recurrent arrhythmias with Mediterranean diet (PREDIMAR) study in patients with atrial fibrillation: rationale, design and methods. Am Heart J.

[120] Pastori D, Carnevale R, Menichelli D, Nocella C, Bartimoccia S, Novo M, et al. Is there an interplay between adherence to Mediterranean diet, antioxidant status, and vascular disease in atrial fibrillation patients?. Antioxid Redox Signal. 2016;25(14):751–5. 10.1089/ars.2016.6839 27577528

[121] Liang Z, Dong Z, Guo M, Shen Z, Yin D, Hu S (2019). Trimethylamine N-oxide as a risk marker for ischemic stroke in patients with atrial fibrillation. J Biochem Mol Toxicol.

[122] Zhu W, Wang Z, Tang WHW, Hazen SL (2017). Gut microbe-generated trimethylamine N-oxide from dietary choline is prothrombotic in subjects. Circulation.

[123] Skye SM, Zhu W, Romano KA, Guo CJ, Wang Z, Jia X (2018). Microbial transplantation with human gut commensals containing cutC is sufficient to transmit enhanced platelet reactivity and thrombosis potential. Circ Res.

[124] Marques FZ, Nelson E, Chu PY, Horlock D, Fiedler A, Ziemann M (2017). High-fiber diet and acetate supplementation change the gut microbiota and prevent the development of hypertension and heart failure in hypertensive mice. Circulation.

[125] Chong E, Chang SL, Hsiao YW, Singhal R, Liu SH, Leha T (2015). Resveratrol, a red wine antioxidant, reduces atrial fibrillation susceptibility in the failing heart by PI3K/AKT/eNOS signaling pathway activation. Heart Rhythm.

[126] Catry E, Bindels LB, Tailleux A, Lestavel S, Neyrinck AM, Goossens JF (2018). Targeting the gut microbiota with inulin-type fructans: preclinical demonstration of a novel approach in the management of endothelial dysfunction. Gut.

[127] Brown GD, Gordon S (2001). Immune recognition. A new receptor for beta-glucans. Nature.

[128] Ryan PM, London LE, Bjorndahl TC, Mandal R, Murphy K, Fitzgerald GF (2017). Microbiome and metabolome modifying effects of several cardiovascular disease interventions in apo-E(-/-) mice. Microbiome.

[129] Zhang Y, Sun D, Zhao X, Luo Y, Yu H, Zhou Y (2022). Bacteroides fragilis prevents aging-related atrial fibrillation in rats via regulatory T cells-mediated regulation of inflammation. Pharmacol Res.

[130] Depommier C, Everard A, Druart C, Plovier H, Van Hul M, Vieira-Silva S (2019). Supplementation with Akkermansia muciniphila in overweight and obese human volunteers: a proof-of-concept exploratory study. Nat Med.

[131] Linz B, Hohl M, Mishima R, Saljic A, Lau DH, Jespersen T (2020). Pharmacological inhibition of sodium-proton-exchanger subtype 3-mediated sodium absorption in the gut reduces atrial fibrillation susceptibility in obese spontaneously hypertensive rats. Int J Cardiol Heart Vasc.

[132] Andraws R, Berger JS, Brown DL (2005). Effects of antibiotic therapy on outcomes of patients with coronary artery disease: a meta-analysis of randomized controlled trials. JAMA.

[133] Hu X, Li H, Zhao X, Zhou R, Liu H, Sun Y (2021). Multi-omics study reveals that statin therapy is associated with restoration of gut microbiota homeostasis and improvement in outcomes in patients with acute coronary syndrome. Theranostics.

[134] Li W, Li C, Ren C, Zhou S, Cheng H, Chen Y (2023). Bidirectional effects of oral anticoagulants on gut microbiota in patients with atrial fibrillation. Front Cell Infect Microbiol.

[135] Orman M, Bodea S, Funk MA, Campo AMD, Bollenbach M, Drennan CL (2019). Structure-guided identification of a small molecule that inhibits anaerobic choline metabolism by human gut bacteria. J Am Chem Soc.

[136] Pathak P, Helsley RN, Brown AL, Buffa JA, Choucair I, Nemet I (2020). Small molecule inhibition of gut microbial choline trimethylamine lyase activity alters host cholesterol and bile acid metabolism. Am J Physiol Heart Circ Physiol.

[137] Wang Z, Roberts AB, Buffa JA, Levison BS, Zhu W, Org E (2015). Non-lethal inhibition of gut microbial trimethylamine production for the treatment of atherosclerosis. Cell.

[138] Smits LP, Kootte RS, Levin E, Prodan A, Fuentes S, Zoetendal EG (2018). Effect of vegan fecal microbiota transplantation on carnitine- and choline-derived trimethylamine-N-oxide production and vascular inflammation in patients with metabolic syndrome. J Am Heart Assoc.

[139] Manrique P, Zhu Y, van der Oost J, Herrema H, Nieuwdorp M, de Vos WM (2021). Gut bacteriophage dynamics during fecal microbial transplantation in subjects with metabolic syndrome. Gut Microbes.

[140] Martin-Cabezas R, Seelam N, Petit C, Agossa K, Gaertner S, Tenenbaum H (2016). Association between periodontitis and arterial hypertension: a systematic review and meta-analysis. Am Heart J.

[141] Schaefer AS, Bochenek G, Jochens A, Ellinghaus D, Dommisch H, Güzeldemir-Akçakanat E (2015). Genetic evidence for PLASMINOGEN as a shared genetic risk factor of coronary artery disease and periodontitis. Circ Cardiovasc Genet.

[142] Chen DY, Lin CH, Chen YM, Chen HH (2016). Risk of atrial fibrillation or flutter associated with periodontitis: a nationwide, population-based, cohort study. PLoS ONE.

[143] Yu G, Yu Y, Li YN, Shu R (2010). Effect of periodontitis on susceptibility to atrial fibrillation in an animal model. J Electrocardiol.

[144] Chang Y, Woo HG, Park J, Lee JS, Song TJ (2020). Improved oral hygiene care is associated with decreased risk of occurrence for atrial fibrillation and heart failure: a nationwide population-based cohort study. Eur J Prev Cardiol.

